# The exquisite link between potassium homeostasis regulation and cardiovascular health: exploration and analysis

**DOI:** 10.3389/fphys.2026.1772535

**Published:** 2026-04-01

**Authors:** Ci Wang, Xiangyuan Huang, Zeyu Zhang, Dongming Lin, Shuwei Huang

**Affiliations:** 1 The First Affiliated Hospital of Zhejiang Chinese Medical University (Zhejiang Provincial Hospital of Chinese Medicine), Hangzhou, China; 2 Zhejiang Chinese Medical University, Hangzhou, China; 3 School of Traditional Chinese Medicine, Beijing University of Chinese Medicine, Beijing, China; 4 First Teaching Hospital of Tianjin University of Traditional Chinese Medicine, National Clinical Research Center of Chinese Medicine Acupuncture and Moxibustion, Tianjin, China; 5 Tianjin University of Traditional Chinese Medicine, Tianjin, China

**Keywords:** arrhythmias, cardiovascular diseases, ion channel, pharmacological strategies, potassium homeostasis

## Abstract

Cardiovascular diseases (CVDs) are the leading global cause of mortality, with potassium homeostasis playing a fundamental role in their pathophysiology. Tightly regulated potassium ions (K^+^) are essential for cardiac electrophysiological stability, and their dysregulation is a critical driver of disorders, particularly cardiac arrhythmias. Systemic potassium homeostasis is maintained by a complex network involving dietary intake, renal and intestinal handling, neuromodulatory control, skeletal muscle buffering and membrane ion channel activity, et al, which together determine extracellular and intracellular potassium homeostasis. This review summarizes the physiological mechanisms underlying potassium homeostasis and critically examines how potassium imbalance contributes to CVDs, with a primary focus on arrhythmia-related pathophysiology. By integrating experimental and clinical evidence, we highlight clinically relevant mechanisms and potential therapeutic strategies aimed at optimizing potassium homeostasis, thereby providing a conceptual framework to improve CVDs prevention and management.

## 1 Introduction

The cardiovascular system, composed of the heart and blood vessels, is responsible for delivering blood, oxygen, and nutrients throughout the body and plays an essential role in maintaining organismal homeostasis (Gao, 2019). Cardiovascular diseases (CVDs) remain the most prevalent diseases and leading causes of mortality worldwide, particularly in developed countries, and their complex etiologies pose a substantial threat to human health (MacRae et al., 2016). According to National Health and Nutrition Examination Survey (NHANES) survey data from 2017 to March 2020, the prevalence of CVDs among adults aged 20 and over in the United States reached 48.6%, with incidence increasing markedly with age (Tsao et al., 2023). Projections estimate that by 2030, more than 23 million people globally will die from CVDs (Mahmood et al., 2014; Amini et al., 2021). The pathogenesis of CVDs is multifactorial and involves diverse mechanisms, including inflammatory responses, oxidative stress, cellular autophagy, and ion channel dysfunction. With rapid advances in modern medicine and deeper investigations at genetic, ionic, and electrophysiological levels, the mechanistic basis of CVDs is increasingly being elucidated.

Cardiac excitation-contraction coupling is initiated by action potentials (APs), which arise from tightly regulated transmembrane ionic fluxes. The precise balance of ions inside and outside the cardiomyocytes is fundamental to maintaining the myocardium’s regular electrical activity and coordinated myocardial contraction. Ionic disturbances are the initiating factors of cardiac electrophysiological disorder and are a significant cause of CVDs. Among all ions, potassium ions (K^+^) constitute the most abundant intracellular cation and play indispensable roles in numerous physiological and metabolic processes. It is essential for maintaining the osmotic pressure across cell membranes, electrophysiological activities, acid-base balance, and neuromuscular function (Kovesdy et al., 2017). K^+^ is involved throughout the resting and excitation states of myocardial cells. In normal circumstances, K^+^ levels in intracellular and extracellular fluids are dynamically balanced, maintaining potassium homeostasis (Macdonald and Struthers, 2004; Kettritz and Loffing, 2023). Imbalances in potassium homeostasis can lead to abnormal distribution of intracellular K^+^ and serum potassium levels, resulting in various diseases. Excessive accumulation, excessive loss, or abnormal distribution of intracellular K^+^ can cause abnormal cardiac electrical activity, making it easier to induce various arrhythmias. Hypokalemia has been strongly associated with the occurrence of diseases like prolonged QT interval, premature ventricular contractions (PVCs), atrial fibrillation (AF), ventricular fibrillation (VF), sudden cardiac death, and hypertension (Castro and Raij, 2013; Krogager et al., 2021). Hyperkalemia can also cause significant cardiac injury by depolarizing the resting membrane potential of cardiomyocytes, inactivating voltage-gated sodium channels, and slowing impulse conduction. In severe cases, sustained conduction failure may culminate in cardiac arrest and sudden death (Rosano et al., 2018). Regulating and maintaining potassium homeostasis is of paramount importance in the prevention and treatment of CVDs.

In recent years, increasing attention has been directed toward potassium regulation, particularly focusing on dietary potassium intake and renal handling of potassium (DuBose, 2017; Kettritz and Loffing, 2023). However, existing studies predominantly emphasize abnormal serum potassium levels, while the broader regulatory network of potassium homeostasis and its mechanistic links to CVDs remain insufficiently explored. In this review, we review and summarize the regulatory mechanisms of potassium homeostasis and the relationship between the imbalance of potassium homeostasis and CVDs. It aims to deepen the understanding of the relationship between potassium homeostasis and CVDs, providing promising insights and directions for future targeted therapies focusing on potassium homeostasis in treating CVDs.

## 2 Regulation mechanism of potassium homeostasis

**Figure 1:**
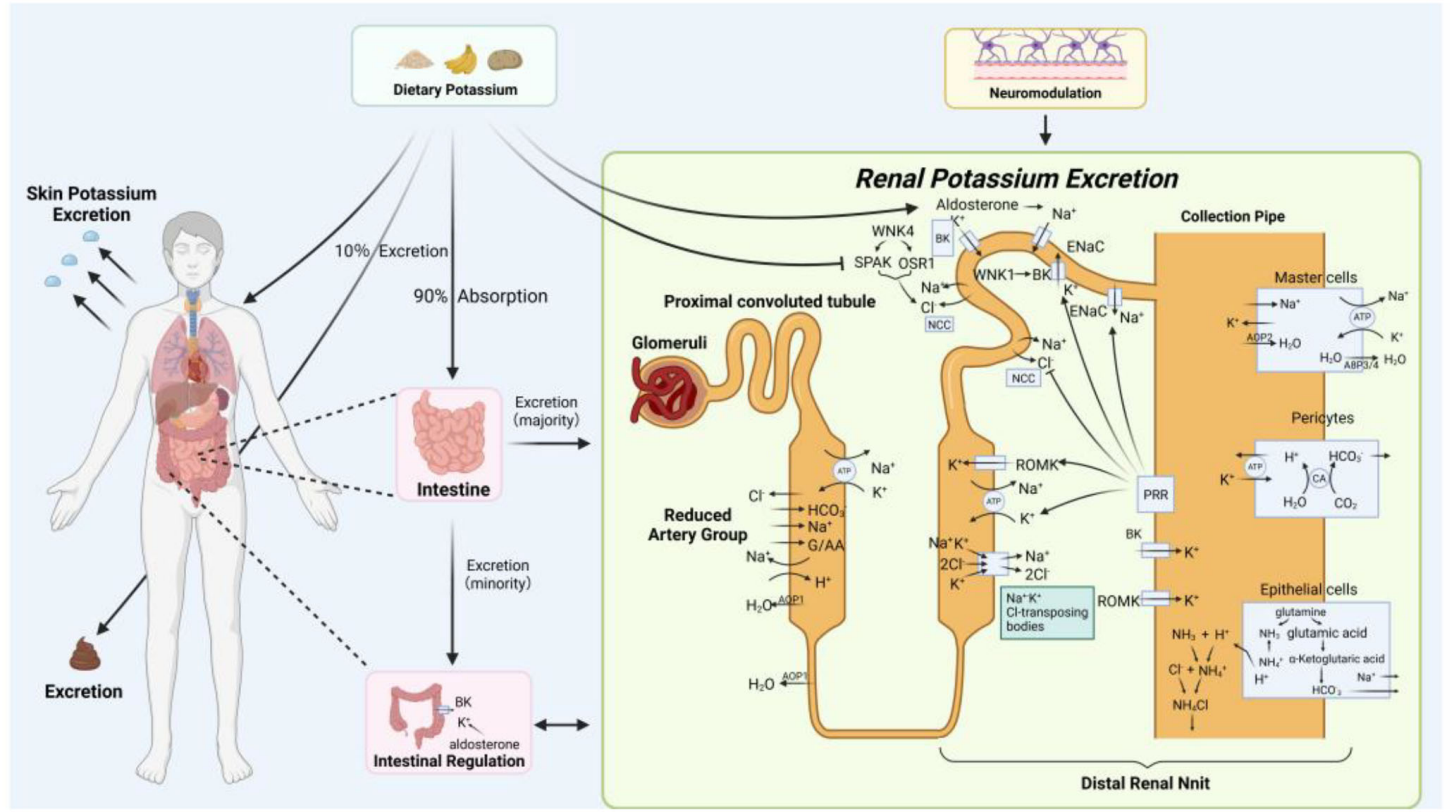
Mechanisms of Body Regulation of Potassium Homeostasis. The regulation and maintenance of potassium homeostasis result from coordinated interactions among dietary intake, gastrointestinal absorption, transcellular redistribution, and renal excretion. Dietary potassium represents the primary source of systemic K^+^. Approximately 90% of ingested potassium is absorbed in the gastrointestinal tract and enters the circulation via the portal vein. Once in the bloodstream, potassium is distributed between the extracellular and intracellular compartments, a process tightly regulated by insulin, catecholamines, and acid–base status. The kidneys serve as the principal organ for long-term potassium balance. Filtered potassium undergoes segment-specific reabsorption and secretion along the nephron, with fine-tuning occurring in the distal tubule and collecting duct. Hormonal regulators, particularly aldosterone, as well as tubular flow rate and sodium delivery, modulate potassium secretion. Approximately 10% of potassium is excreted through feces and sweat. Skeletal muscle and the nervous system further contribute to rapid buffering and redistribution of potassium, ensuring stable extracellular potassium concentrations.

**Figure 2:**
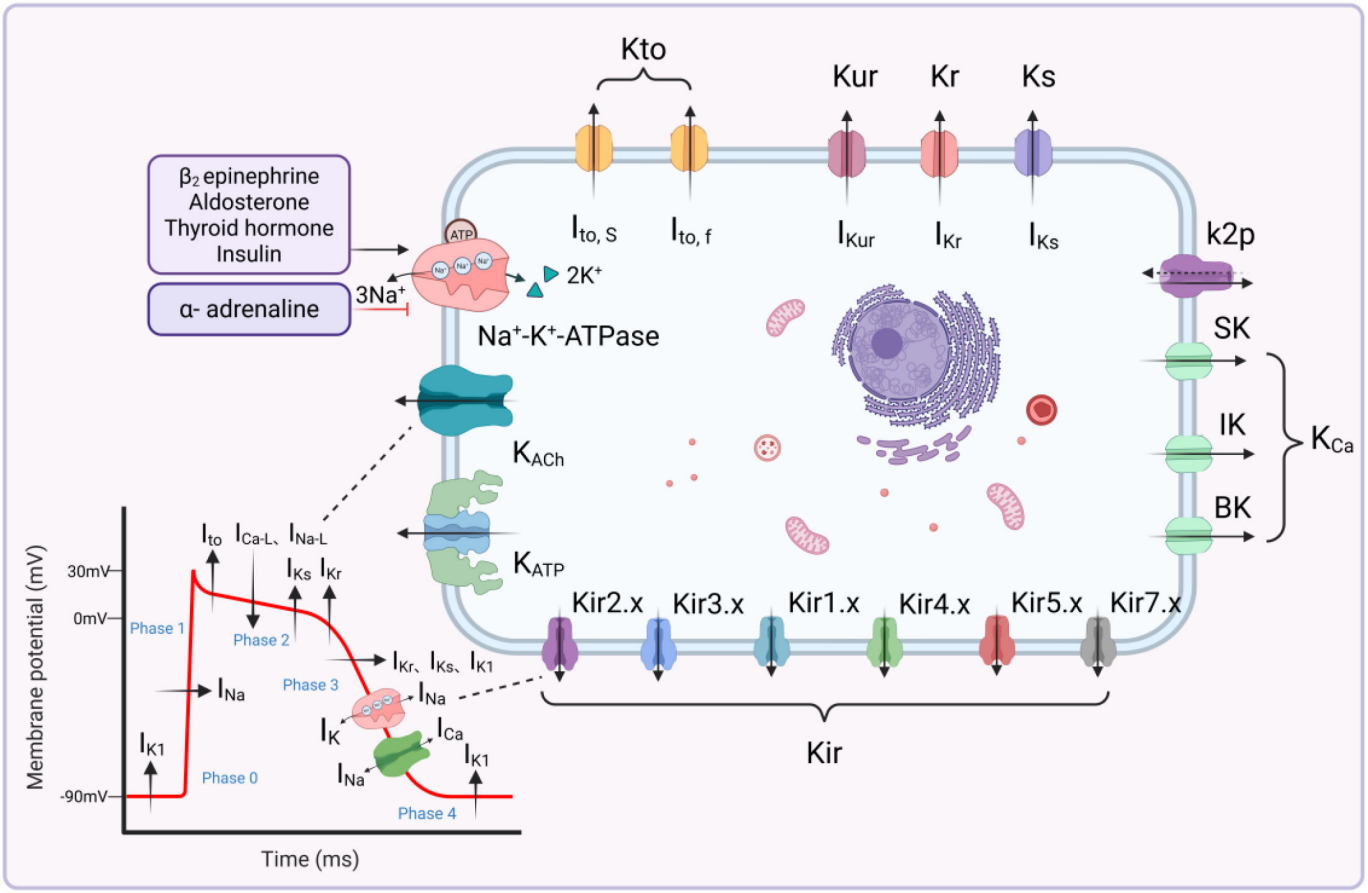
Ionic Regulatory Mechanisms of Intracellular Potassium Homeostasis. K^+^ are involved in and maintain both the resting potential and the entire action potential of cardiomyocytes. Intracellular potassium homeostasis is primarily regulated by the dynamic opening and closing of potassium channels. The coordinated activity of sodium, potassium, and calcium channels together shapes the complete action potential of the cardiomyocyte. At rest (Phase 4), the membrane potential is primarily maintained by the I_K1_, which permits outward K^+^ flux and stabilizes the resting membrane potential close to the potassium equilibrium potential. During rapid depolarization (Phase 0), voltage-gated sodium channels (I_Na_) open, resulting in a rapid influx of Na^+^. Although most potassium channels remain closed at this stage, the transmembrane K^+^ gradient critically determines cellular excitability and depolarization threshold. In early repolarization (Phase 1), I_to_ mediates brief K^+^ efflux, producing partial membrane repolarization. The plateau phase (Phase 2) is characterized by a balance between inward I_Ca-L_, I_Kr_ and I_Ks_. This dynamic equilibrium maintains sustained depolarization and supports excitation–contraction coupling. During final repolarization (Phase 3), enhanced activation of I_Kr_ and I_Ks_, together with reactivation of I_K1_, promotes K^+^ efflux and restores the membrane potential toward resting levels. Intracellular potassium homeostasis is further maintained by the Na^+^/K^+^-ATPase, which actively transports K^+^ into the cell and Na^+^ out of the cell, preserving the transmembrane ionic gradients. In addition, the Na^+^/Ca²^+^ exchanger (NCX) contributes to ionic balance by extruding Ca²^+^ in exchange for Na^+^, indirectly influencing intracellular sodium concentration and thereby affecting Na^+^/K^+^-ATPase activity and overall potassium homeostasis. The coordinated activity of potassium, sodium, and calcium channels and transporters ensures stable cardiac electrophysiological function.

Potassium homeostasis refers to the dynamic equilibrium of K^+^ levels inside and outside cells. Potassium homeostasis is characterized by the balance between K^+^ intake and excretion in the body, normal plasma K^+^ levels, stable concentrations of K^+^ inside and outside cells, and stable interactions between K^+^ and other ions (Palmer and Clegg, 2019). The human body employs various regulatory mechanisms to maintain potassium homeostasis. External potassium homeostasis depends mainly on dietary potassium intake, renal regulation, intestinal regulation, neural regulation, and skeletal muscle regulation. Internal potassium homeostasis is primarily regulated by genes, hormones, and ion channels on cell membranes (**Figures 1**, **2**).

### 2.1 Ion channels

From a physiological perspective, different potassium channel subtypes contribute sequentially to distinct phases of the cardiac action potential, thereby shaping electrocardiographic features. Potassium channels represent the most abundant and structurally diverse class of ion channels in the heart. Composed of pore-forming subunits arranged around a central conduit and often associated with auxiliary subunits, these channels are distributed across the sarcolemma and organelle membranes of cardiomyocytes, where they critically regulate resting membrane potential and action potential duration (APD). Based on their gating mechanisms, potassium channels are broadly categorized into voltage-gated (K_v_) and ligand-gated types. Voltage-gated potassium channels include the transient outward (K_to_), delayed rectifier, and inwardly rectifying (Kir) subtypes, whereas ligand-gated channels comprise ATP-sensitive (K_ATP_) and acetylcholine-sensitive (K_ACh_) potassium channels (Tamargo et al., 2004). By controlling the direction and magnitude of potassium flux, sarcolemmal potassium channels play a vital role in maintaining cellular potassium homeostasis.

Kto consists of Kv1.4, Kv4.2, and Kv4.3, and is present in the atria, ventricles, and cardiac conduction system. The transient outward potassium current (I_to_) is the predominant current during the early repolarization phase of myocardial cells, categorized into two functional phenotypes: I_to,f_ and I_to,s_. Rapid activation and inactivation of I_to_ form phase 1 of the cardiac AP, mediating potassium efflux and contributing to forming the early repolarization phase and regulating the AP plateau voltage level (He et al., 2015).

Delayed rectifier potassium channels are categorized into three subtypes: fast delayed rectifier potassium channel (Kr), slow delayed rectifier potassium channel (Ks), and ultra-rapid delayed rectifier potassium channel (Kur). Kur is primarily distributed in atrial muscle cells and is mainly composed of the Kv1.5α subunit encoded by KCNA5. The ultra-rapid delayed rectifier potassium current (I_Kur_) features rapid activation and slow deactivation, primarily contributing to atrial repolarization. Kr is composed of Kv11.1 (also known as the human ether-a-go-go-related gene (hERG), while Ks is primarily made up of the KCNQ1 (Kv7.1) and KCNE1 subunits. The slow delayed rectifier potassium current (I_Ks_) activates very slowly during the AP formation and is responsible for phase 3 repolarization (Chen et al., 2016). When the intracellular K^+^ concentration increases, the activity of delayed rectifier potassium channels increases, promoting the efflux of K^+^ and thus maintaining intracellular potassium homeostasis.

Kir comprises seven subfamilies, grouped into four functional categories: classical Kir (Kir2.x), G-protein gated Kir (Kir3.x), K_ATP_ (Kir6.x), and potassium transport channels (Kir1.x, Kir4.x, Kir5.x, and Kir7.x). Given their diverse structures and types, Kir is involved in regulating multiple physiological functions such as neuronal signal transmission, heart rate, blood flow, and insulin secretion (Bichet et al., 2003; Hibino et al., 2010). The inward-rectifying characteristic of Kir helps prevent excessive K^+^ efflux, which is essential for maintaining internal potassium homeostasis.

K_ATP_, a part of the Kir family, consists of four K^+^-conducting subunits (Kir6.2) and four sulfonylurea receptor (SUR)2A subunits, which confer sensitivity to sulfonylureas and potassium channel openers on channel (Foster and Coetzee, 2016). Normally, intracellular ATP acts as an inhibitor of K_ATP_. K_ATP_ bridges cellular metabolism and membrane excitability, orchestrating various physiological functions. In the event of myocardial ischemia, ATP depletion activates K_ATP_, suggesting that the channel serves a protective function in ischemia-reperfusion (I/R) scenarios (Aziz et al., 2017).

K_ACh_ consists of two distinct inward-rectifying potassium channel subunits and a newly cloned family member, CIR, acting as a regulatory target for the autonomic nervous system and adenosine. It is primarily located in pacemaking tissues (sinoatrial node (SAN), atrioventricular node, and Purkinje fibers) and atrial muscle, involved in parasympathetic regulation of heart rate (Krapivinsky et al., 1995).

In addition to the aforementioned potassium channels, two-pore-domain potassium channels (K_2_P) and calcium-activated potassium channels (K_Ca_) are newly discovered channels in cardiac cells, playing significant roles in various biological activities. K_2_P consists of 15 subunits, each dimer formed by four transmembrane helices (M1-M4) and two pore domains (P1-P2). K_2_P generates almost instantaneous and non-inactivating currents within the range of membrane potentials, exhibiting unique electrophysiological properties, and plays a significant role in cardiac repolarization and the development of various arrhythmias (Wiedmann et al., 2021). K_2_P exhibits both outwardly rectifying characteristics and, under certain conditions, weak inwardly rectifying properties. This bidirectional transport capability allows for precise regulation of intracellular and extracellular K^+^ concentrations, playing a unique role in maintaining potassium homeostasis. K_Ca_ is a class of potassium channels that is sensitive to both voltage and Ca^2+^. They are categorized into three main subfamilies based on their single-channel conductance: small conductance calcium-activated potassium channels (SK), intermediate conductance calcium-activated potassium channels (IK), and big conductance calcium-activated potassium channels (BK). When intracellular calcium ion concentration increases, K_Ca_ is activated to mediate K^+^ efflux, participating in the regulation of potassium homeostasis by reducing intracellular potassium concentration and affecting cell excitability and neurotransmitter transmission (Brown et al., 2020; Orfali and Albanyan, 2023) (**Table 1**).

**Table 1:** Major potassium channels involved in cardiac potassium homeostasis.

Potassium channel (current)	Major cardiac location	Primary physiological function	Representative encoded genes
Transient outward potassium channel (I_to_)	Atria, ventricles, cardiac conduction system	Mediates early repolarization (phase 1) of cardiac action potential; regulates plateau voltage	*KCND3* (Kv4.3), *KCND2* (Kv4.2), *KCNA4* (Kv1.4)
Ultra-rapid delayed rectifier potassium channel (I_Kur_)	Atrial cardiomyocytes	Contributes to atrial repolarization; shortens atrial action potential duration	*KCNA5* (Kv1.5)
Rapid delayed rectifier potassium channel (I_Kr_)	Atria and ventricles	Major determinant of phase 3 repolarization; critical for action potential duration and QT interval	*KCNH2* (hERG, Kv11.1)
Slow delayed rectifier potassium channel (I_Ks_)	Atria and ventricles	Facilitates phase 3 repolarization, especially during sympathetic stimulation	*KCNQ1* (Kv7.1), *KCNE1*
Inward rectifier potassium channel (I_K1_)	Ventricular cardiomyocytes	Maintains resting membrane potential; stabilizes terminal repolarization	*KCNJ2* (Kir2.1)
ATP-sensitive potassium channel (I_KATP_)	Sarcolemma of cardiomyocytes	Couples cellular metabolic state to membrane excitability; protective during ischemia	*KCNJ11* (Kir6.2), *ABCC9* (SUR2A)
Acetylcholine-sensitive potassium channel (I_KACh_)	Sinoatrial node, atrioventricular node, atria	Mediates parasympathetic regulation of heart rate; slows pacemaker activity	*KCNJ3* (Kir3.1), *KCNJ5* (Kir3.4)
Two-pore-domain potassium channels (I_K2P_)	Atria and ventricles	Contribute to background K^+^ conductance; modulate repolarization and arrhythmogenesis	*KCNK* family (e.g., *KCNK3*, *KCNK9*)
Calcium-activated potassium channels (I_KCa_)	Cardiomyocytes and cardiac-related excitable cells	Activated by intracellular Ca²^+^; regulate excitability and K^+^ efflux	*KCNN* (SK), *KCNA1* (IK), *KCNMA1* (BK)

hERG, human ether-a-go-go-related gene; SK, Small conductance calcium-activated potassium channel; IK, Intermediate conductance calcium-activated potassium channel; BK, Big conductance calcium-activated potassium channel; Kv, Voltage-gated potassium channel; Kir, Inwardly rectifying potassium channel; SUR, Sulfonylurea receptor.

### 2.2 Dietary potassium

Dietary potassium serves as the primary source of K^+^ for the body and is essential for maintaining potassium homeostasis. Potassium is widely present in foods such as fruits (e.g., bananas, oranges), vegetables, legumes, and dairy products (**Table 2**) (Rizzoli, 2014; Czech et al., 2020; McLean and Wang, 2021; Abate et al., 2024). In nutritional studies, its intake is often estimated via dietary recalls or, more objectively, assessed through 24-hour urinary potassium excretion, which correlates closely with recent consumption (Gamba and Ellison, 2025). Extensive epidemiological and clinical evidence indicates that adequate potassium intake confers multiple health benefits, including lowered blood pressure, reduced cardiovascular mortality, slower progression of kidney disease, decreased diabetes incidence, and improved bone health (He and MacGregor, 2008; Stone et al., 2016). According to U.S. dietary guidelines, the recommended daily intake is 2600 mg for adult women and 3400 mg for men; however, only about 2% of adults meet these recommendations (Cogswell et al., 2012; Bailey et al., 2021).

**Table 2:** Potassium-rich foods and their potassium content.

Food Category	Food Name	Potassium Content per 100g
Fruits	Bananas	350 mg
	Oranges	145 mg
Vegetables	Potatoes	600 mg
	Spinach	530 mg
Legumes	Fava beans	228–236 mg
	Chickpea	581–881 mg
Dairy Products	Milk	151–156 mg
	Yogurt	216–234 mg

In cardiovascular health, potassium intake helps improve endothelial function—an early marker of cardiovascular risk (D’Elia et al., 2023). In terms of blood pressure regulation, the antihypertensive effect of adequate dietary potassium is primarily mediated through elevated plasma K^+^ levels. An acute rise in plasma K^+^ stimulates potassium channels on vascular smooth muscle cells, promoting arterial dilation and lowering blood pressure. Additionally, K^+^ modulates baroreceptor sensitivity, reducing responsiveness to pressor neurotransmitters such as catecholamines (Haddy et al., 2006). Additionally, dietary potassium promotes dephosphorylation of the sodium-chloride cotransporter (NCC) via PP1A, counteracting the WNK/SPAK signaling cascade that increases sodium reabsorption (Grimm et al., 2023). The mechanisms underlying this process are described in more detail in Section 2.4.2. Nevertheless, high potassium intake is not universally advisable and should be restricted in patients with chronic kidney disease, hyperkalemia, or those on hemodialysis (Ramos et al., 2021; Sun et al., 2023).

Conversely, inadequate intake compromises intestinal barrier integrity, increasing permeability and risk of bacterial translocation, which may lead to diarrhea, malnutrition, or spontaneous bacterial peritonitis (Wu et al., 2022). Low potassium intake also reduces insulin sensitivity and elevates diabetes risk, in part through activation of the WNK/SPAK-NCC pathway (Ekmekcioglu et al., 2016).

Therefore, maintaining dietary potassium within a physiologically appropriate range-neither deficient nor excessive-is crucial for preserving potassium homeostasis and supporting overall health, with intake tailored to individual clinical conditions (Little et al., 2023).

### 2.3 Hormonal regulation

Hormonal regulation can regulate potassium homeostasis by affecting K^+^ transmembrane transport, with Na^+^-K^+^-ATPase (NKA) playing a crucial role in this process. NKA is present in almost all cells, using the energy released from hydrolyzing one ATP molecule to transport 3 Na^+^ out of the cell and pump 2 K^+^ in. This reverse concentration transport mechanism is crucial for maintaining the intracellular fluid distribution balance of potassium. Insulin, catecholamines, thyroid hormones, and β2 receptor agonists can enhance NKA activity, participating in the regulation of potassium homeostasis.

Insulin is the primary hormone affecting K^+^ transmembrane transport. Eating stimulates insulin secretion, which not only regulates glucose levels but also increases NKA activity, transferring K^+^ to muscle and other tissue cells before renal excretion, thus reducing the renal burden of potassium excretion. Furthermore, high extracellular K^+^ concentration also stimulates insulin secretion to maintain potassium homeostasis inside and outside the cells. Therefore, glucose-insulin-potassium therapy not only regulates blood glucose homeostasis but also maintains potassium homeostasis inside and outside the cells. This therapy inhibits the synthesis of pro-inflammatory cytokines and promotes the synthesis of endothelial nitric oxide and anti-inflammatory cytokines, proving effective in CVDs such as myocardial infarction (Das, 2002; Udelson et al., 2022).

Furthermore, catecholamines, thyroid hormones, and aldosterone are also important hormones influencing K^+^ transmembrane transport. Increased potassium intake can promote catecholamine secretion, which binds to β2-adrenergic receptors to stimulate K^+^ entering skeletal muscle cells and binds to α-adrenergic receptors to move K^+^ to extracellular spaces (Rosa et al., 1980; DeFronzo et al., 1981). Besides increasing NKA activity, thyroid hormones can shorten APD by upregulating left ventricular Kv1.5 mRNA levels, accelerating the cardiac repolarization process (Nishiyama et al., 1997). Aldosterone plays an essential role in the hormonal and renal regulation of potassium homeostasis. High serum potassium levels stimulate the adrenal gland to secrete aldosterone, which increases NKA activity to transport K^+^ into cells, maintaining the balance of K^+^ inside and outside the cells. Aldosterone’s renal regulation mainly manifests in its promotion of renal potassium excretion function, as detailed in the following section.

### 2.4 Renal regulation

Healthy kidneys possess a robust ability to excrete potassium, which is essential in maintaining potassium homeostasis. Once K^+^ enters the kidneys, they are freely filtered in the glomerulus. Subsequently, 90% of potassium is reabsorbed in the proximal tubule and thick ascending limb of the loop of Henle, with final secretion and reabsorption occurring in the distal nephron. Renal potassium excretion mainly relies on the aldosterone-sensitive distal nephron (ASDN), which consists of the late distal convoluted tubule (DCT), connecting tubule (CNT), and cortical collecting duct (CCD) (Rodan, 2017). The regulation of renal potassium excretion by the distal nephron plays an important role in maintaining the body’s potassium homeostasis.

#### 2.4.1 The role of aldosterone in renal regulation

Aldosterone, being a crucial hormone for potassium homeostasis regulation, plays roles in both hormonal and renal aspects of potassium regulation. In the distal nephron, aldosterone binds to mineralocorticoid receptors in principal cells, upregulating and activating the apical epithelial Na^+^ channel (ENaC). Enhanced Na^+^ reabsorption through ENaC generates a lumen-negative transepithelial potential difference, which provides the electrochemical driving force for potassium secretion through apical potassium channels, particularly the renal outer medullary potassium channel (ROMK) (Wu et al., 2020; Nesterov et al., 2022). In addition, increased intracellular Na^+^ stimulates basolateral NKA activity, which extrudes Na^+^ from the cell in exchange for K^+^ entry. This process raises intracellular K^+^ concentration, further supporting sustained potassium secretion into the tubular lumen.

Under physiological conditions of high dietary potassium intake, aldosterone-independent mechanisms may also contribute to potassium secretion (Todkar et al., 2015). In the DCT and early CNT, the infusion of high potassium or angiotensin II (Ang II) activates ENaC-mediated Na^+^ intake, providing the electrochemical driving force for ROMK-mediated tubular K^+^ secretion to excrete excess potassium, independent of aldosterone.

#### 2.4.2 Potassium homeostasis regulation of distal nephron

K^+^ transport in the distal nephron is regulated in a segment-specific manner, primarily within the DCT and the ASDN, which includes the CNT and CCD. Within these segments, coordinated activity of transporters and channels such as NCC, ENaC, ROMK, and BK determines the balance between sodium reabsorption and potassium secretion. Salt reabsorption in the DCT is mediated by the thiazide-sensitive NCC. NCC is a transmembrane protein, 1002 to 1030 amino acids long, that reabsorbs 5%-10% of filtered NaCl in the nephron’s DCT (Gamba, 2023). The WNK/SPAK/odd-skipped related 1 (OSR1) signaling pathway is crucial for activating NCC and maintaining potassium homeostasis. The WNK family comprises four genes: WNK1 to WNK4. WNK1 and WNK3 are expressed in all nephron segments, whereas WNK4 is mainly found in aldosterone-sensitive nephron units (Castañeda-Bueno et al., 2022). WNK kinases phosphorylate and activate the downstream kinases SPAK and OSR1, which in turn directly phosphorylate and activate NCC, thereby enhancing NaCl reabsorption in the DCT. In high-potassium conditions, the WNK/SPAK/OSR1 signaling pathway inhibits NCC’s NaCl reabsorption in the DCT, directing Na^+^ to the ASDN. In the ASDN, enhanced ENaC sodium reabsorption creates a luminal negative charge, driving potassium excretion in an aldosterone-dependent manner. In low-potassium conditions, NCC-mediated NaCl reabsorption in the DCT is enhanced (Castañeda-Bueno et al., 2014). Along the nephron, potassium secretion is subsequently mediated by apical potassium channels that are differentially expressed in specific tubular segments, ensuring segment-specific regulation of potassium handling.

ROMK and BK are the principal apical potassium channels mediating potassium secretion in distinct segments of the distal nephron. ROMK, encoded by the KCNJ1 gene, serves as the main channel for potassium secretion under normal potassium intake conditions. ROMK is predominantly expressed in the thick ascending limb (TAL) and the apical membrane of principal cells in the CNT and CCD, where it provides basal potassium secretion under physiological conditions. ROMK forms the main apical membrane conductance in the TAL, facilitating the K^+^ efflux transport by Na^+^-K^+^-2Cl^-^ cotransporter 2 (NKCC2) (Welling and Ho, 2009). Following a high-potassium diet, aldosterone can activate ENaC, providing the driving force for ROMK-mediated potassium secretion.

BK is composed of a pore-forming α subunit and a regulatory β subunit. In contrast to ROMK, BK channels are primarily localized to the CNT and CCD and are activated under conditions of increased tubular flow or high potassium intake, providing an adaptive pathway for potassium secretion. The α subunit is associated with flow-induced renal potassium secretion and plays a potential role in potassium homeostasis regulation (Latorre et al., 2017). BK expression is markedly upregulated with increased potassium intake. BK function is influenced by transient receptor potential vanilloid type 4 (TRPV4) channels and WNKs. TRPV4 is a Ca^2+^-permeable channel, highly expressed in the kidneys, and determines flow-dependent intracellular Ca^2+^ increase. Studies have found that TRPV4 channels couple with BK function, and their functional loss leads to decreased BK activity and reduced potassium excretion capacity (Mamenko et al., 2017; Stavniichuk et al., 2023). WNK1 enhances BK function by inhibiting extracellular signal-regulated kinase (ERK) 1/2-mediated channel lysosomal degradation, while WNK4 inhibits BK activity by promoting channel lysosomal degradation (Wang et al., 2013; Liu et al., 2015).

Moreover, the (pro)renin receptor ((P)RR) participates in the regulation of potassium homeostasis by activating the local renin-angiotensin-aldosterone system (RAAS) and stimulating K^+^ secretion in the distal nephron. At the cellular level, (P)RR is predominantly expressed on the apical membrane of principal cells in the distal nephron. (P)RR is highly expressed in the connecting tubule (CNT) and cortical collecting duct (CCD), and high potassium loads upregulate the expression of (P)RR and aldosterone in the kidney. Conversely, hypokalemia enhances (P)RR-mediated signaling by activating the intrarenal RAAS, thereby modulating downstream transporters involved in potassium conservation. (P)RR significantly inhibits NCC activity by increasing the cleavage product, soluble (P)RR from site-1 protease, and enhances the levels of β-ENaC, ROMK, α-BK, and α-NKA, potentially playing a role in the renal regulation of potassium homeostasis (Xu et al., 2017; Xu C. et al., 2021). Through this pathway, (P)RR links potassium deficiency to coordinated regulation of sodium reabsorption and potassium secretion in the distal nephron (**Table 3**).

**Table 3:** Potassium transport and regulatory mechanisms along the distal nephron.

Nephron segment mechanisms	Major transporters channels	Potassium handling	Key regulatory
TAL	NKCC2, ROMK (KCNJ1)	K^+^ reabsorption	Luminal K^+^ recycling via ROMK
DCT	NCC	Indirect regulation of K^+^ excretion	WNK–SPAK–OSR1 signaling pathway
CNT	ENaC, ROMK, BK	K^+^ secretion	Aldosterone, tubular flow
CCD	ENaC, ROMK, BK	K^+^ secretion/fine-tuning	Aldosterone, (P)RR, WNK signaling

TAL, Thick ascending limb; NKCC2, Na^+^-K^+^-2Cl⁻ cotransporter 2; ROMK, Renal outer medullary K^+^ channel; DCT, Distal convoluted tubule; NCC, Sodium-chloride cotransporter; WNK, With no lysine (K) kinase; SPAK, STE20/SPS1-related proline/alanine-rich kinase; OSR1, Odd-skipped related 1; CNT, Connecting tubule; ENaC, Epithelial Na^+^ channel; CCD, Cortical collecting duct; (P)RR, (Pro)renin receptor.

### 2.5 Intestinal regulation

K^+^ is primarily absorbed in the intestine. 90% of the potassium ingested by the body is absorbed by the intestines, with the remainder excreted in the feces. The potassium absorbed in the intestines first enters the intestinal epithelial cells and is subsequently primarily filtered out and expelled by the kidneys. Therefore, the intestines and kidneys act in coordination to regulate potassium balance, which is essential for maintaining potassium homeostasis under both physiological and pathological conditions.

The absorption of potassium in the intestines is primarily based on apical membrane mechanisms and paracellular passive diffusion, allowing K^+^ to enter the bloodstream via the portal circulation (Agarwal et al., 1994). Increasing evidence supports the existence of an intestinal–renal potassium signaling axis, through which dietary potassium intake directly influences renal potassium excretion. Specifically, intestinal peptides and other humoral factors released in response to dietary potassium intake can target tissue kallikrein and NADPH oxidase in the kidney, thereby modulating potassium secretion in the distal nephron. Through this mechanism, increased dietary potassium intake promotes renal potassium excretion independently of changes in serum potassium levels. In addition, a small proportion of potassium can be excreted directly through BK channels in the colon, a process that is aldosterone-dependent (Sørensen et al., 2010; Nickerson and Rajendran, 2021).

Even in pathological conditions, the intestines adaptively regulate K^+^. During chronic renal failure, the potassium secretion capacity of the rectal mucosa gradually increases, which helps the body quickly achieve a new homeostasis and potassium homeostasis under pathological conditions (Martin et al., 1986).

### 2.6 Neuroregulation

Accumulating evidence indicates that the nervous system directly participates in the regulation of potassium homeostasis. The proposed hepatic reflex theory suggests that potassium-sensitive receptors located in the liver or hepatic portal circulation can detect dietary potassium intake and reflexively promote potassium excretion via vagal afferent signaling. Additionally, studies indicate that infusing hypertonic NaCl into the third ventricle can also reflexively increase potassium excretion. Pituitary gland removal surgery reduces or weakens these two reflex responses (Rabinowitz and Aizman, 1993).

The nervous system significantly impacts the skeletal muscle regulation of potassium homeostasis and can provide an effective buffer when the body experiences a substantial loss of potassium. In a rat model of hypertension induced by potassium depletion, hypothalamic centers suppress the α-adrenergic activity in slow muscle fibers and an unidentified humoral factor in fast muscle fibers, thereby inhibiting the activity of the skeletal muscle NKA and preventing further loss of K^+^.

The kidney’s regulation of potassium homeostasis shows a distinct circadian rhythm, providing evidence of the nervous system’s role in potassium regulation. Kidney regulation of potassium follows a circadian rhythm characterized by reduced excretion at night and in the morning, and increased excretion in the afternoon, which aligns with the circadian rhythm of renal potassium transporter expression. In extrarenal tissues, the inflow and outflow of potassium follow a circadian cycle, with the peaks of potassium outflow matching those of renal excretion. The periodic regulation of K^+^ uptake and excretion by tissues may originate from an oscillator in the hypothalamus, but the specific location of the oscillator and the nature of the signals it emits are not yet clear (Rabinowitz, 1996; Gumz and Rabinowitz, 2013). The mechanisms through which the nervous system regulates potassium homeostasis remain to be thoroughly investigated.

### 2.7 Skeletal muscle regulation

As the largest organ for potassium storage, skeletal muscle plays a critical role in buffering extracellular potassium fluctuations. Its excitability depends on the expression and coordinated activity of multiple potassium transporters, allowing skeletal muscle to dynamically regulate the distribution of K^+^ between intracellular and extracellular compartments. During normal physical activity, intracellular K^+^ shifts into the skeletal muscle interstitium, transiently increasing local potassium concentration and inducing vasodilation to meet metabolic demands. Conversely, during fasting or low-potassium intake, skeletal muscle can release stored potassium into the extracellular fluid to prevent excessive declines in serum K^+^ levels (McDonough and Youn, 2005).

Four major potassium transport proteins participate in skeletal muscle potassium handling: NKA, inward rectifier potassium channels (Kir), Kir6.2, and Na^+^-K^+^-2Cl^-^ cotransporter 1 (NKCC1). Kir channels facilitate potassium efflux and contribute to the stabilization of the resting membrane potential and repolarization of action potentials. Kir6.2, a metabolically sensitive Kir channel, links intracellular ATP levels to membrane excitability. In resting mixed-fiber skeletal muscles, NKA and NKCC1 mediate partial potassium influx, which is crucial for maintaining normal muscle function. NKA accounts for approximately 50% of potassium influx, whereas NKCC1 contributes about 12%, particularly under conditions of osmotic stress, where it participates in cell volume regulation. Collectively, these transport systems enable skeletal muscle to serve as a dynamic potassium reservoir during exercise, fasting, and dietary potassium fluctuations. Insufficient potassium intake during intense physical activity may impair skeletal muscle perfusion and, in severe cases, contribute to rhabdomyolysis (Palmer, 2015; Lindinger and Cairns, 2021).

Importantly, skeletal muscle buffering precedes renal potassium excretion following food intake. Postprandial insulin release stimulates skeletal muscle and hepatic potassium uptake, thereby reducing the immediate renal excretory burden and stabilizing extracellular potassium levels. In patients with end-stage renal disease, dietary potassium restriction during the interdialytic period further highlights the essential buffering role of skeletal muscle in systemic potassium homeostasis (**Tables 4**, **5**).

**Table 4:** Skeletal muscle potassium handling mechanisms.

Transporter/Channel	Direction of K^+^ flux	Major physiological role	Key regulatory factors (as described)	Physiological/pathological relevance
Na^+^/K^+^-ATPase (NKA)	Influx	Maintains resting membrane potential; mediates major K^+^ uptake in skeletal muscle	Insulin; neural regulation	Accounts for ~50% of K^+^ influx; contributes to postprandial K^+^ buffering and prevention of extracellular hypokalemia
Inward rectifier K^+^ channels (Kir)	Efflux	Stabilizes resting membrane potential; contributes to action potential repolarization	Membrane voltage	Maintains skeletal muscle excitability
Kir6.2 (K_ATP_ channel)	Efflux	Links metabolic state to membrane potential by sensing intracellular ATP	Intracellular ATP concentration	Enables adaptation to metabolic stress
Na^+^-K^+^-2Cl^-^ cotransporter 1 (NKCC1)	Influx	Contributes to K^+^ influx; involved in cell volume regulation	Osmotic stress/cell swelling	Mediates ~12% of K^+^ influx; may participate in skeletal muscle responses during stress or exercise

**Table 5:** Extrarenal regulation of potassium homeostasis.

Regulatory system	Primary stimulus	Sensor/initiating site	Major effector organ	Proposed role in potassium homeostasis
Intestinal regulation	Dietary potassium intake	Intestinal epithelium/portal circulation	Kidney	Activates the intestinal–renal potassium excretion axis via intestinal peptides and fluid factors, promoting renal K^+^ excretion
Neuroregulation	Increased dietary K^+^; central osmotic stimuli	Hepatic/portal potassium-sensitive receptors; hypothalamus	Kidney; skeletal muscle	Reflexively enhances potassium excretion and modulates skeletal muscle K^+^ retention
Skeletal muscle buffering	Insulin release; neural signals	Skeletal muscle fibers	Skeletal muscle	Acts as a dynamic reservoir to buffer extracellular K^+^ fluctuations, especially after meals or during potassium depletion

### 2.8 The influence of other ions

Sodium, hydrogen, and magnesium ions all interact with K^+^, participating in the regulation of potassium homeostasis by influencing the activity of potassium channels and potassium transport.

Na^+^ is crucial for the regulation of potassium homeostasis. Many physiological functions of K^+^, such as those involving NKA and NKCC on the cell membrane, are co-regulated by both Na^+^ and K^+^. Both Na^+^ and K^+^ are involved in regulating blood pressure, and decreasing Na^+^ intake along with increasing potassium intake can contribute to lowering blood pressure (Whelton, 2014). Additionally, in the renal regulation of potassium homeostasis, ENaC-mediated Na^+^ reabsorption is closely coupled with K^+^ excretion. In the DCT, sodium handling directly influences downstream potassium secretion. At the level of the distal convoluted tubule, sodium–potassium interactions are further coordinated through NCC-mediated sodium handling. The phosphorylation of NCC induced by Ang II infusion can be reversed by increased potassium intake (Wen et al., 2014a; Veiras et al., 2016), thereby reducing NaCl reabsorption in the DCT, increasing sodium delivery to the aldosterone-sensitive distal nephron, and ultimately promoting renal potassium excretion.

Hydrogen and K^+^ collaboratively sustain the body’s acid-base equilibrium. Disruptions in this balance greatly impact transmembrane transport and renal excretion of K^+^. In metabolic acidosis, increased extracellular hydrogen ion concentration promotes H^+^ entry into cells in exchange for K^+^ efflux across the cell membrane, which may result in hyperkalemia. At the renal level, enhanced hydrogen ion secretion occurs primarily in the distal nephron, including the distal convoluted tubule and collecting duct. This process is accompanied by increased potassium reabsorption mediated mainly by H^+^-K^+^-ATPase in α-intercalated cells, contributing to elevated serum potassium levels. In cases of metabolic alkalosis, a reduction in body hydrogen ion concentration prompts cells to absorb K^+^ and expel hydrogen ions, resulting in hypokalemia. In the distal nephron, reduced hydrogen ion secretion is associated with decreased H^+^-K^+^-ATPase activity and diminished potassium reabsorption, thereby exacerbating urinary potassium loss and hypokalemia (Kellum, 2005).

Magnesium ions are critical for energy storage and utilization, serving as necessary components of numerous enzyme systems (Souza et al., 2023). Magnesium ions activate NKA, which enhances the influx of K^+^ and inhibits their efflux, thereby acting as inward rectifiers and regulating the balance of K^+^ across cell membranes. Magnesium ion deficiency is associated with heart failure (HF) and various arrhythmias. This may be related to changes in the resting membrane potential caused by alterations in intracellular and extracellular K^+^ concentrations (Wester, 1992).

## 3 The imbalance of potassium homeostasis

Potassium homeostasis is defined by a steep transmembrane gradient, with the vast majority of total body potassium located intracellularly and only a small fraction present in the extracellular fluid. This distribution is essential for maintaining membrane potential and electrical activity in excitable tissues, particularly the heart. As a result, extracellular potassium concentration must be tightly regulated within a narrow physiological range. Even minor fluctuations in serum potassium levels can markedly alter cellular excitability and electrical conduction, leading to severe cardiovascular and neuromuscular consequences (Binaghi et al., 2025).

### 3.1 Abnormal serum potassium

#### 3.1.1 Hyperkalemia

A serum potassium level exceeding 5.5mmol/L indicates hyperkalemia, representing a significant sign of extracellular fluid potassium imbalance. Common causes of hyperkalemia include increased potassium intake, decreased renal potassium excretion, and transcellular potassium shifts, with renal excretion disturbances being the primary factor (Nilsson et al., 2017). Patients with acute or chronic renal failure and those experiencing significant blood pressure drop often face reduced glomerular filtration rates and increased blood potassium levels. Patients with renal diseases are also prone to hyperkalemia when there is a reduction in intestinal potassium excretion. This reduction is partly attributed to impaired colonic potassium secretion in chronic kidney disease, which is associated with decreased aldosterone responsiveness and altered activity of aldosterone-sensitive potassium channels, particularly BK channels in the colon. Aldosterone deficiency or impaired mineralocorticoid signaling, resulting from adrenal insufficiency or pharmacological inhibition, can disrupt potassium secretion in the distal tubules and collecting ducts, leading to hyperkalemia. Utilization of RAAS inhibitors (RAASi) and potassium-sparing diuretics significantly increases the risk of hyperkalemia by inhibiting aldosterone’s effects on sodium retention and potassium excretion, causing potassium retention (Larivée et al., 2023). Abnormal transcellular potassium transport is a common pathogenic mechanism of hyperkalemia, typically seen in conditions such as metabolic acidosis, cellular destruction, tissue hypoxia, and hyperkalemic periodic paralysis. Mechanical injuries like hemolysis and crush injuries associated with rhabdomyolysis can lead to cellular destruction and consequently, a significant release of potassium from cells. During tissue hypoxia, reduced ATP production impairs the transport function of the NKA, decreasing the transport of K^+^ into cells. Moreover, hyperkalemic periodic paralysis, a group of hereditary diseases characterized by intermittent muscle weakness and paralysis, also presents with hyperkalemia during episodes (Statland et al., 2018). This condition is most commonly caused by mutations in skeletal muscle ion channels, particularly voltage-gated sodium channels such as SCN4A, which alter membrane excitability and potassium handling.

Another hereditary disorder associated with hyperkalemia is Gordon syndrome, also known as pseudohypoaldosteronism type II (PHAII). Gordon syndrome is characterized by hypertension, hyperkalemia, and metabolic acidosis and is caused by mutations in genes encoding components of the WNK signaling pathway, including WNK1, WNK4, KLHL3, and CUL3. These mutations enhance NCC activity in the distal convoluted tubule, leading to increased NaCl reabsorption and reduced sodium delivery to the aldosterone-sensitive distal nephron. Consequently, ENaC-mediated sodium reabsorption and potassium secretion are diminished, resulting in hyperkalemia (Mabillard and Sayer, 2019).

Hyperkalemia suppresses the automaticity, conductivity, and contractility of the heart muscle, initially enhancing and subsequently inhibiting myocardial excitability. Hyperkalemia is associated with poor outcomes in CVDs, leading to cardiac conduction block and, in severe cases, can cause cardiac arrest. High potassium levels not only compromise the normal physiological functions of the heart but also interfere with normal nerve and skeletal muscle cell conduction. Patients may initially experience abnormal limb sensations, numbness, and muscle soreness, progressing to muscle weakness or paralysis in severe cases. Elevated serum potassium levels induce metabolic acidosis by altering renal ammonia metabolism, which further enhances the exchange between extracellular potassium and intracellular hydrogen ions, aggravating hyperkalemia and causing significant harm to the body (Cook et al., 2021).

#### 3.1.2 Hypokalemia

Hypokalemia is defined as a serum potassium concentration below 3.5 mmol/L. This condition commonly leads to impaired glucose tolerance, cardiac damage, renal damage, gastrointestinal injury, skeletal muscle damage, and neurological dysfunction (Nilsson et al., 2017).

Decreased potassium intake, excessive renal potassium excretion, gastrointestinal losses, or transcellular shifts are the main causes of hypokalemia (Greenlee et al., 2009). Hypokalemia resulting from reduced potassium intake is commonly seen in patients who are unable to eat or can only consume small amounts of food. Excessive renal potassium excretion is primarily seen in patients with renal tubular acidosis, magnesium deficiency, and those using potassium-wasting diuretics, with the latter being the most common cause.

In addition to acquired causes, several hereditary renal tubular disorders lead to inappropriate renal potassium wasting. Liddle syndrome is caused by gain-of-function mutations in ENaC subunits, resulting in enhanced sodium reabsorption in the collecting duct, increased lumen-negative potential, and excessive potassium secretion (Tetti et al., 2018). Bartter syndrome arises from defects in transporters of the thick ascending limb, including NKCC2, ROMK, or ClC-Kb, leading to salt wasting, secondary hyperaldosteronism, and increased distal potassium secretion (Rodríguez-Soriano, 1998). Gitelman syndrome is caused by loss-of-function mutations in NCC in the distal convoluted tubule, reducing sodium reabsorption and enhancing aldosterone-mediated potassium excretion (Blanchard et al., 2017).

Magnesium deficiency promotes renal potassium wasting primarily by disinhibiting ROMK channels in the distal nephron. Intracellular magnesium normally suppresses ROMK channel activity; therefore, hypomagnesemia enhances ROMK-mediated potassium secretion, leading to refractory hypokalemia (Yang et al., 2010). Although magnesium may also influence NKA, increased ROMK activity represents the predominant mechanism underlying hypokalemia in magnesium deficiency. The most frequent clinical cause of hypokalemia is gastrointestinal loss, especially from severe diarrhea, frequent vomiting, and high-output gastrointestinal fistulas (such as enterocutaneous or biliary fistulas), where significant amounts of potassium are lost with digestive fluids. Hypokalemia due to abnormal potassium transport across cells is often associated with metabolic alkalosis, specific toxic exposures(such as barium or chloroquine),or hypokalemic periodic paralysis. Excessive use of insulin or β-adrenergic agonists overstimulates NKA activity, increasing potassium transport into cells and reducing extracellular potassium levels, resulting in hypokalemia (Weiner and Wingo, 1997; Kim et al., 2023).

Hypokalemia heightens the excitability and automaticity of heart muscle cells, reduces their conductivity, and leads to an initial increase followed by a decrease in myocardial contractility. Hypokalemia readily leads to various cardiac arrhythmias and heightens the sensitivity of HF patients to the toxic effects of digitalis (Weiss et al., 2017). Hypokalemia suppresses insulin secretion, resulting in impaired glucose tolerance (Zillich et al., 2006). Patients with hypokalemia experience reduced gastrointestinal function, commonly manifesting as poor appetite and indigestion, with severe cases leading to paralytic ileus. Sustained hypokalemia impairs renal tubular responsiveness to antidiuretic hormone, leading to a functional defect in urinary concentrating ability and resulting in polyuria and nocturia characteristic of nephrogenic diabetes insipidus. In severe cases of hypokalemia, insufficient energy in the muscles leads to ischemia and hypoxia, which can cause muscle cramps, necrosis, and rhabdomyolysis. Hypokalemia can also cause neurological dysfunction, with severe cases leading to symptoms such as somnolence or even coma.

### 3.2 Abnormal intracellular potassium concentration

As the predominant cation within an intracellular fluid, K^+^ is critical for maintaining its volume and osmotic pressure. The K^+^ concentration in the intracellular fluid is largely determined by the collective impact of various potassium channels and the NKA on the cell membrane. Damage to outward potassium channels such as Kto, K_Ca_, and delayed rectifier potassium channels, or overactivation of Kir, can lead to a decrease in potassium efflux and an abnormal increase in intracellular K^+^ concentration. Conversely, when outward potassium channels are overactivated or Kir is impaired, potassium efflux increases, resulting in an abnormal decrease in intracellular K^+^ concentration. The bidirectional transport properties of K_2_P uniquely contribute to the regulation of K^+^ currents. Furthermore, NKA is crucial in influencing the distribution of K^+^ across cell membranes, with its activity affected by multiple factors such as hormones, neurotransmitters, sodium ion concentration, and energy supply. Increased activity of NKA facilitates the influx of K^+^, raising K^+^ concentrations within the intracellular fluid, whereas decreased activity lowers these concentrations.

In conclusion, disruption of potassium homeostasis within intracellular fluid can alter cell morphology and membrane potential, thereby impacting normal cellular functions. An imbalance in intracellular potassium also disrupts external potassium homeostasis, which can lead to hypokalemia or hyperkalemia, adversely affecting human health.

## 4 The imbalance of potassium homeostasis and CVDs

**Figure 3:**
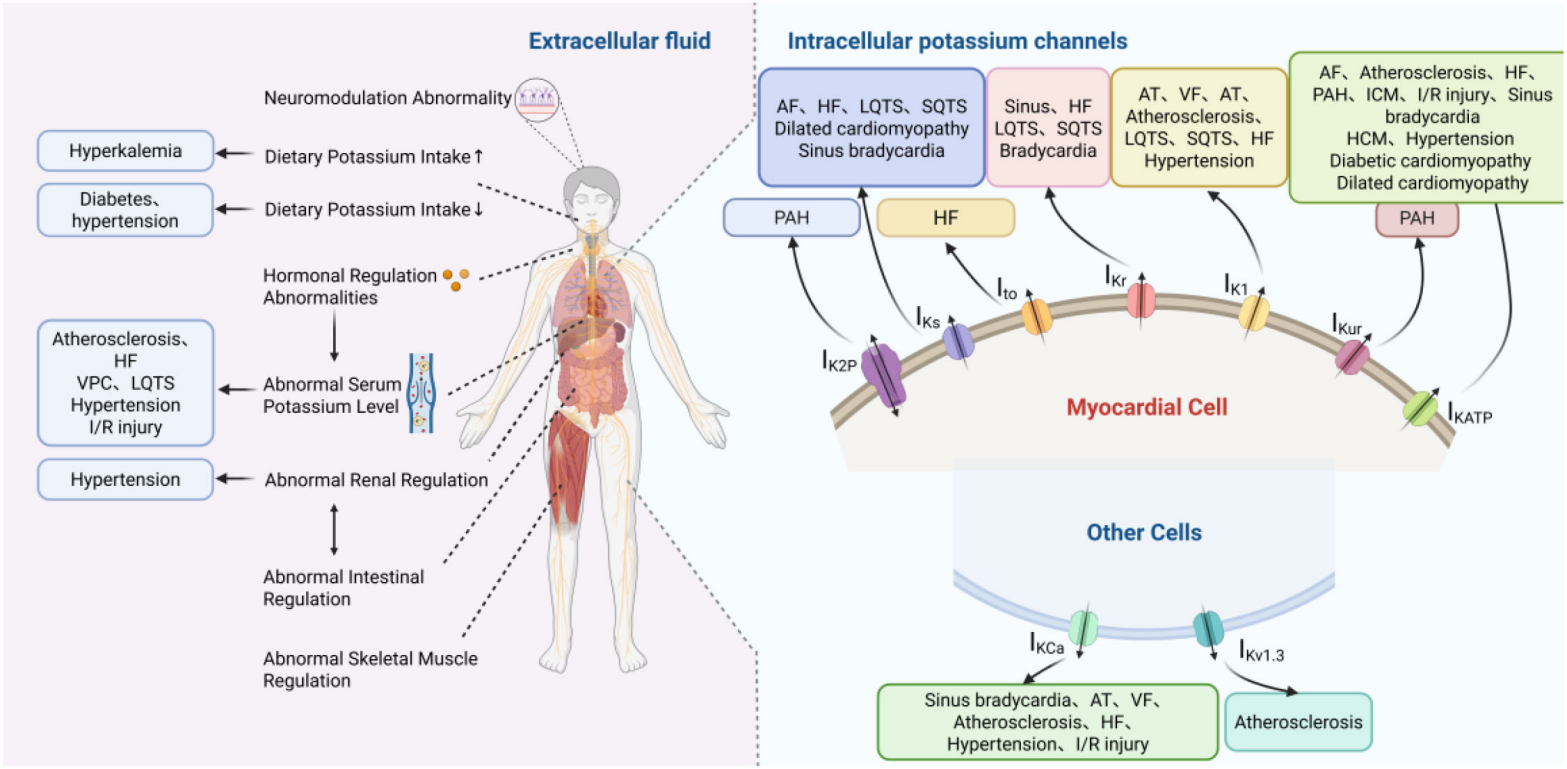
The relationship between the imbalance of potassium homeostasis and cardiovascular diseases. Potassium homeostasis imbalance involves disturbances in both intracellular potassium distribution and extracellular (serum) potassium concentration. Intracellular K^+^ dysregulation is commonly associated with genetic mutations of potassium channel–encoding genes, altered channel gating, or abnormal expression and trafficking of ion channels, leading to disrupted membrane excitability and action potential configuration. Changes in transmembrane K^+^ gradients directly affect resting membrane potential, action potential duration, and repolarization stability. Disturbances in blood potassium levels (hyperkalemia or hypokalemia) arise from multiple systemic factors, including dietary intake, gastrointestinal absorption, renal excretion, hormonal regulation (e.g., aldosterone), and acid–base status. Because extracellular K^+^ concentration determines the electrochemical gradient across the cell membrane, fluctuations in serum potassium dynamically influence intracellular potassium distribution and cellular excitability. Disruption of potassium homeostasis is strongly associated with cardiovascular diseases. Abnormal K^+^ levels can alter myocardial conduction velocity and repolarization reserve, thereby promoting arrhythmogenesis. Sustained imbalance contributes to structural and electrical remodeling, increasing the risk of heart failure (HF), arrhythmias, hypertension, pulmonary arterial hypertension (PAH), long QT syndromes (LQTs), and short QT syndromes (SQTS).

Disruption of potassium homeostasis increases the risk of developing and dying from multiple CVDs (**Figure 3**). Hence, maintaining potassium stability is critically important for CVDs prevention (Fan et al., 2024).

### 4.1 Arrhythmias

#### 4.1.1 Atrial fibrillation

Atrial fibrillation, the most common clinical arrhythmia, presents with symptoms such as palpitations, shortness of breath, and syncope. It can also be disabling or even fatal (Brundel et al., 2022). Its prevalence increases annually with age, posing a significant public health challenge. The clinical outcomes of AF are complex. Recurrent AF often leads to atrial electrical remodeling and changes in atrial myocyte ion channels, primarily manifesting as shortened effective refractory period (ERP) and APD in electrophysiological terms (Schotten et al., 2011).

Imbalances in intracellular and extracellular potassium homeostasis increase susceptibility to AF. Changes in potassium channels within the intracellular fluid play a crucial role in atrial electrical remodeling (Feghaly et al., 2018). These potassium channel-mediated alterations collectively shorten atrial APD and effective refractory period, thereby promoting electrical remodeling and increasing susceptibility to AF (**Table 6**).With the irreversible progression of atrial structures, AF tends to become permanent (Lozano-Velasco et al., 2020). SK channels are abundantly expressed in the atria, and their dysregulation further contributes to AF vulnerability (**Table 6**) (Heijman et al., 2023; Liu et al., 2023). Hypokalemia is associated with an increased risk of AF, and thus maintaining normal serum potassium concentrations is crucial for AF prevention (Farah et al., 2021).

**Table 6:** Potassium channels and mechanisms involved in AF.

Potassium channel/gene	Affected current	Functional change	Electrophysiological consequence	Role in AF
KCNQ1 (Kv7.1)	I_Ks_	Gain-of-function	↑ I_Ks_, shortened APD and ERP	Promotes atrial electrical remodeling and reentry
KCNH family (Kv11.x)	I_Kr_/I_to_	Increased outward currents	Accelerated repolarization	Facilitates AF susceptibility
KCNA5 (Kv1.5)	I_Kur_	Abnormal channel function	Shortened atrial APD	Contributes to atrial remodeling
KCNJ2 (Kir2.1)	I_K1_	Gain-of-function	↑I_K1_, hyperpolarized membrane	Stabilizes reentry circuits
KCNJ8 (Kir6.1)	IK_ATP_	Gain-of-function	↑ I_KATP_	Enhances AF vulnerability
SK channels	I_SK_	Overactivation or inhibition	APD shortening or instability	Increases AF susceptibility
Hypokalemia	Multiple K^+^ currents	Reduced extracellular K^+^	Enhanced atrial excitability	Clinical risk factor for AF

AF, Atrial fibrillation; APD, Action potential duration; ERP, Effective refractory period; SK, Small conductance calcium-activated potassium channels.

#### 4.1.2 Premature contraction

Depending on the location of the ectopic beat, Premature contractions can be classified into atrial premature contractions, junctional premature contractions, and PVCs. PVCs are particularly common and exhibit a wide variety of clinical symptoms, with some patients experiencing palpitations, chest discomfort, and sensations of skipped heartbeats (Hoogendijk et al., 2020). Potassium homeostasis is involved in the development of PVCs, with research indicating that lowered serum potassium levels are linked to a higher incidence of PVCs (Tsuji et al., 1994). Disturbances in potassium homeostasis increase myocardial excitability and electrical heterogeneity, thereby predisposing to premature contractions (Podrid, 1990) (**Table 7**).

**Table 7:** Potassium homeostasis and premature contractions (PACs, PVCs).

Factor	Associated change	Electrophysiological effect	Contribution to premature contractions
Hypokalemia	↓ Serum K^+^	↑ Automaticity and excitability	Increases ectopic firing
Altered K^+^ currents	APD and AERP prolongation	Abnormal ventricular conduction	Facilitates PVC occurrence
Potassium imbalance	Membrane instability	Triggered activity	Higher incidence of PVCs

PACs, Premature atrial contractions; PVCs, Premature ventricular contractions; AERP, Atrial effective refractory period.

#### 4.1.3 Sinus bradycardia

Sinus bradycardia is a cardiac arrhythmia where the SAN exhibits reduced automaticity, leading to a sinus rhythm of less than 60 beats per minute. Often resulting from insufficient blood supply to the SAN or pathological changes in nearby tissues, sinus bradycardia can cause hemodynamic abnormalities and underperfusion of critical organs like the heart, brain, and kidneys, typically manifesting in sick sinus syndrome (Kusumoto et al., 2019). Dysfunctions in potassium channels like K_ACh_, Kr, Ks, and SK are involved in the development of sinus bradycardia. Hyperpolarization-activated cyclic nucleotide-gated 4 (HCN4) is the predominant subtype of the HCN protein family in the SAN (Verkerk and Wilders, 2014). It forms ion channels that generate the funny current (I_f_) significantly regulating the SAN’s pacemaker activity. Overexpression of K_ACh_ leading to automatic remodeling of the SAN can suppress hyperpolarization-activated HCN4 channels and I_f_, thereby increasing the likelihood of training-induced sinus bradycardia (Bidaud et al., 2020). Selective deletion of ERG1 B eliminates the rapidly deactivating component of I_Kr_ in fetal cardiac myocytes and all I_Kr_ in adult ventricular myocytes, thereby triggering episodes of sinus bradycardia (Lees-Miller et al., 2003). Altered potassium channel activity in the sinoatrial node suppresses pacemaker automaticity and contributes to sinus bradycardia (**Table 8**) (Whittaker et al., 2018; Wan et al., 2019).

**Table 8:** Potassium channels involved in sinus bradycardia.

Channel/gene	Current	Functional alteration	Effect on SAN activity	Clinical relevance
K_ACh_	I_KACh_	Overexpression	Suppressed pacemaker activity	Training-induced sinus bradycardia
HCN4	I_f_	Functional inhibition	Reduced automaticity	Core mechanism of SAN slowing
ERG1 (KCNH2)	I_Kr_	Loss of function	Prolonged repolarization	Sinus bradycardia episodes
KCNQ1	I_Ks_	Gain-of-function	Reduced pacing rate	Genetic sinus bradycardia
SK channels	I_SK_	Inhibition	↓ Atrial and ventricular automaticity	Bradyarrhythmia susceptibility

K_ACh_, Acetylcholine-sensitive potassium channel; HCN4, Hyperpolarization-activated cyclic nucleotide-gated 4; I_f_, Funny current; SAN, Sinoatrial nodel; I_SK_, SK current.

#### 4.1.4 Ventricular tachycardia and VF

VT is defined as a sustained or non-sustained ventricular arrhythmia originating from the ventricles, characterized by three or more consecutive ventricular beats at a rate exceeding 100 beats per minute (AlMahameed and Ziv, 2019). VT may degenerate into VF. Potassium imbalance increases the risk of VT and VF and leads to poor outcomes. Thus, maintaining stable potassium levels is crucial in the treatment strategies for VT and VF (Schupp et al., 2020).

Potassium currents such as I_K1_ and SK current (I_SK_) can disrupt potassium homeostasis, inducing the occurrence and development of VT and VF. Mutations in KCNJ2 can lead to the loss of adrenergic-dependent I_K1_ during the final repolarization phase and trigger phase 3 early afterdepolarizations (EADs), thereby inducing VT (Chun et al., 2004; Reilly et al., 2020). This stabilization of re-entry rotors consequently increases susceptibility to VF (Jalife, 2009). Abnormal potassium currents promote ventricular electrical instability, facilitating reentrant activity and increasing susceptibility to VT and VF (**Table 9**) (Yin et al., 2017).

**Table 9:** Potassium currents in VT and VF.

Potassium current/channel	Pathological change	Electrophysiological effect	Arrhythmogenic mechanism
I_K1_	Loss or gain of function	EADs or rotor stabilization	VT induction or VF maintenance
SK current (I_SK_)	Overactivation	Shortened APD	Sustained VF
Potassium imbalance	Serum K^+^ instability	Conduction abnormalities	Poor VT/VF outcomes

VT, Ventricular tachycardia; VF, Ventricular fibrillation; EADs, Early afterdepolarizations.

#### 4.1.5 Long QT syndrome and short QT syndrome

Dysfunction of potassium channels is often caused by genetic defects, disease-related tissue remodeling, or adverse drug reactions. LQTS and SQTS are two of the most common potassium channelopathies (Burg and Attali, 2021).

LQTS is a familial genetic disorder associated with cardiac repolarization dysfunction, characterized by prolonged QT intervals and abnormal ECG T-waves, typically associated with torsades de pointes (TdP) and sudden cardiac death. LQTS often manifests as recurrent fainting episodes, and in severe instances, can result in life-threatening cardiac arrest (Krahn et al., 2022). The pathogenesis of LQTS involves an imbalance in intracellular and extracellular potassium homeostasis, including abnormalities in potassium channels and serum potassium levels.

From a mechanistic perspective, LQTS is primarily caused by a reduction in outward repolarizing potassium currents, resulting in delayed ventricular repolarization and QT interval prolongation. In contrast, SQTS arises from enhanced potassium currents that accelerate repolarization, leading to a shortened QT interval. The major potassium channels and representative genetic alterations underlying these syndromes are summarized in **Table 10** (Zareba and Cygankiewicz, 2008; Schwartz et al., 2012). Moreover, hypokalemia significantly contributes to the risk of LQTS by indirectly lengthening the QTc interval (>460 ms) through impacts on potassium channels. Keeping serum potassium within normal limits is vital for LQTS prevention.

**Table 10:** Potassium channelopathies in LQTS and SQTS.

Syndrome	Channel/gene	Affected current	Functional alteration	QT interval effect
LQTS	KCNQ1	I_Ks_	Loss of function	QT prolongation
	KCNH2	I_Kr_	Loss of function	QT prolongation
	KCNE1/KCNE2	I_Ks_/I_Kr_	Reduced current amplitude	QT prolongation
	KCNJ2	I_K1_	Reduced inward rectification	QT prolongation
SQTS	KCNH2	I_Kr_	Gain of function	QT shortening
	KCNQ1	I_Ks_	Gain of function	QT shortening
	KCNJ2	I_K1_	Gain of function	QT shortening

LQTS, Long QT syndrome; SQTS, Short QT syndrome.

SQTS is a severe and rare genetic heart condition characterized by a shortened QT interval. Typically, there are no significant structural heart abnormalities, but it is prone to episodes of AF or sudden cardiac death. SQTS is predominantly associated with gain-of-function alterations in potassium channels, leading to increased outward potassium currents, abbreviated action potential duration, and a shortened QT interval (**Table 10**) (Bjerregaard, 2018; Hancox et al., 2023).

### 4.2 Atherosclerosis

Atherosclerosis is a chronic vascular inflammatory disease initiated by endothelial dysfunction, followed by lipid deposition and fibrous tissue proliferation within the arterial intima. Common risk factors for atherosclerosis include hypertension, diabetes, smoking, and obesity (Xu S. et al., 2021; Perrotta, 2023). Emerging evidence suggests that disturbances in potassium-related signaling, including dysfunction of potassium channels and insufficient dietary potassium intake, contribute to endothelial dysfunction and vascular remodeling during atherosclerosis development.

Potassium channels are crucial for vascular tone regulation. Dysfunctions in potassium channels like Kv1.3, K_ATP_, K_Ca_3.1 (KCNN4), and Kir2.1 can disturb internal potassium homeostasis, leading to the onset and progression of atherosclerosis. Kv1.3 channels are upregulated in macrophages within atherosclerotic lesions, implicating their involvement in lesion-associated inflammatory responses. Functionally, enhanced Kv1.3 activity promotes ERK and NF-κB signaling in response to oxidized low-density lipoprotein (ox-LDL), thereby amplifying macrophage-driven inflammatory cascades. The gathering and penetration of inflammatory cells directly correlate with macrophage accumulation at plaque sites and the formation scope of atherosclerotic lesions. Therefore, inhibiting Kv1.3 may become a potential therapeutic approach for atherosclerosis (Kan et al., 2016; Zhang et al., 2022). K_ATP_, through their Kir6.1 subunits, can reduce the formation of atherosclerotic plaques and protect endothelial cells (Li et al., 2020). In contrast, in macrophages, KATP channel activation has been reported to facilitate inflammatory signaling via MAPK/NF-κB pathways, thereby accelerating lesion progression (Ling et al., 2013). Macrophages often undergo phenotypic polarization under specific microenvironmental conditions and signaling stimuli to perform distinct functions. Polarized macrophages are classified into classically activated macrophages, which guide pro-inflammatory responses, and alternatively activated macrophages, which drive immune modulation and tissue remodeling. During disease progression, increased K_Ca3.1_ activity in endothelial cells and vascular smooth muscle cells (VSMCs) promotes a pro-inflammatory macrophage phenotype, contributing to plaque instability (Mantovani et al., 2004). Activation of K_Ca_3.1 in macrophages boosts the accumulation of ox-LDL, which triggers the STAT3/CD36 axis to elevate the expression of pro-inflammatory cytokines (Zhu et al., 2019). Therefore, inhibiting these channels could contribute to the stabilization of atherosclerotic plaques (Jiang et al., 2022). Kir2.1-mediated membrane hyperpolarization facilitates calcium influx and ox-LDL uptake in macrophages, thereby supporting foam cell formation and atherogenesis (Zhang et al., 2016).

Insufficient dietary potassium intake may modulate vascular cell behavior and increase susceptibility to atherosclerotic changes. With low potassium intake, enhanced calcium signaling, autophagy, and cAMP response element-binding protein signaling in VSMCs induce osteogenic differentiation and calcification of VSMCs, accelerating arterial calcification in atherosclerosis. Increasing dietary potassium intake can reduce vascular calcification and stiffness, thereby inhibiting the proliferation and migration of VSMCs (Sun et al., 2017).

### 4.3 Heart failure

HF is a clinical syndrome resulting from impaired ventricular systolic or diastolic function. Clinically, patients present with fatigue, dyspnea, and lower limb edema. Severe or terminal CVDs often progress to HF, posing a substantial public health burden (Baman and Ahmad, 2020). Alterations in serum potassium levels and potassium channel function contribute to HF pathogenesis by affecting myocardial excitability, electrical stability, and structural remodeling.

Both hypokalemia and hyperkalemia can adversely influence HF progression through distinct electrophysiological and pharmacological mechanisms. Persistent hypokalemia and transient hyperkalemia are independent predictors of 12-month mortality within hospitals (Caravaca Perez et al., 2022). Hypokalemia increases the automaticity and excitability of cardiac myocytes and enhances the toxicity of digitalis drugs, which are associated with increased incidence and mortality rates of HF (Sfairopoulos et al., 2021). The lower the K^+^ concentration, the higher the risk. Hyperkalemia not only limits the use of RAASi but also can lead to cardiac conduction block and re-entry, thereby promoting the onset of HF (Sarwar et al., 2016). Therefore, maintaining potassium levels within the physiological range is an important consideration in HF management. At the cellular level, disturbances in potassium homeostasis contribute to HF progression by altering cardiac excitability, APD, calcium handling, and myocardial remodeling.

Abnormal potassium channels such as Kto, K1, Ks, Kr, K_ATP_, and SK are involved in the occurrence and development of HF. HF is characterized by coordinated remodeling of multiple potassium currents, including marked reductions in I_to_ and I_K1_, along with moderate downregulation of I_Ks_ and I_Kr_ (Husti et al., 2021). The reduction in these K^+^ currents can promote the occurrence of EADs by prolonging the APD, thus increasing the risk of arrhythmias and exacerbating HF. Myocardial hypertrophy is an early compensatory manifestation of HF, and continuous myocardial hypertrophy can result in the onset of HF. Reduced Ito has also been linked to CaMKII activation and intracellular calcium overload, thereby contributing to maladaptive myocardial hypertrophy (Zicha et al., 2004; Anderson et al., 2011). K_ATP_ may be a potential therapeutic target for HF as it could increase the risk of HF by downregulating serum apolipoprotein A-I levels and activating CaMKII (Gao et al., 2016; Liu et al., 2021). The impact of SK channel activation in HF is highly context-dependent, varying with disease stage and the underlying electrophysiological substrate. Activation of SK in failing hearts can promote the repolarization of myocardial cells and shorten the APD, exerting an anti-arrhythmic effect by reducing triggered activity. However, it can also cause QT interval prolongation and increase the risk of TdP (Chang and Chen, 2015). Therefore, the effects of SK channel activation in HF are highly dependent on the underlying electrophysiological substrate and disease stage.

### 4.4 Hypertension

Hypertension is characterized primarily by increased systemic arterial blood pressure and may be accompanied by functional or organic damage to important organs such as the heart, brain, and kidneys (Byrd and Brook, 2019). It is a significant risk factor for various CVDs and chronic kidney disease. Potassium homeostasis imbalance is crucial in hypertension and its cardiovascular sequelae (Burnier and Damianaki, 2023).

Insufficient potassium intake and renal regulation disorders of potassium homeostasis play important roles in the pathogenesis of hypertension. A cross-sectional study of healthy American adults over the age of 40 revealed an inverse relationship between dietary potassium consumption and pulse pressure, suggesting that a moderate increase in potassium intake could serve as a preventative measure against high blood pressure (Xie et al., 2023). When potassium intake is insufficient, vascular smooth muscle contracts, aldosterone secretion is suppressed, the WNK/SPAK pathway is activated, and a combination of mechanisms leads to increased blood pressure. When renal potassium handling is impaired, dysregulation of tubular ion transport leads to increased sodium reabsorption and reduced sodium excretion, thereby contributing to hypertension. Conversely, hypertension can also trigger hyperkalemia by disrupting renal regulation. Some antihypertensive drugs, such as RAASi, suppress aldosterone secretion and reduce renal potassium excretion, thereby increasing blood potassium levels and impairing kidney function (Mutig and Bachmann, 2019). In patients with hypertension, the elevated blood pressure, along with associated neuroendocrine activation, disrupts the function of renal tubular potassium channels such as ENaC, ROMK, and BK. Specifically, the activation of the RAAS and other neurohormonal pathways contributes to impaired potassium handling in the kidneys, exacerbating potassium retention (Wang et al., 2013; Wen et al., 2014b; Suzumoto et al., 2023). In the long term, microvascular and interstitial kidney damage caused by hypertension further exacerbates this channel dysfunction. Together, these processes impair renal potassium excretion and lead to elevated blood potassium levels.

Additionally, the abnormal opening of some endothelial potassium channels also plays a role in the pathogenesis of hypertension. Inhibition of K_ATP_ in endothelial cells can induce constriction of small arteries, leading to hypertension in rats (Long et al., 2008). In mice with genetic deficiencies in SK3 and I_K1_, the vasorelaxation mediated by an endothelium-derived hyperpolarizing factor is suppressed, leading to increased arterial blood pressure, while the activation of SK3 and I_K1_ reverses this process (Brähler et al., 2009).

### 4.5 Pulmonary arterial hypertension

PAH, a serious cardiopulmonary disorder with various etiologies, is characterized by symptoms such as dyspnea, fatigue, palpitations, and lower limb edema (Galiè et al., 2016). In the most critical cases, it can lead to fatal outcomes. The main pathological features of PAH are vascular remodeling and distal pulmonary artery obstruction. The former is primarily mediated by the excessive proliferation of pulmonary arterial smooth muscle cells (PASMC). The expression and functional homeostasis of potassium channels significantly impact PASMC proliferation and migration, thereby influencing the progression of PAH.

The KCNK3 channel is typically expressed in human PASMC and is crucial for regulating pulmonary vascular tension. Its loss of function is a hallmark of PAH-associated right ventricular hypertrophy and dysfunction (Lambert et al., 2018). Mutations in the KCNK3 gene that result in a loss of function can cause pulmonary artery contraction by inducing depolarization of the resting membrane potential. These mutations are also associated with increased expression of inflammatory mediators such as monocyte chemoattractant protein-1, chemokine growth-regulated oncogene 1, and interleukin-17, which have been implicated in PAH pathogenesis (Antigny et al., 2016). Overexpression of KCNK1 and KCNK2 has also been implicated in the development of PAH. It promotes pulmonary vascular remodeling by enhancing PASMC proliferation and migration via Ca²^+^ signaling and JNK activation (Shima et al., 2024).

K_ATP_ channels have been reported to modulate PASMC proliferation and vascular function. SUR1-dependent K_ATP_ activators can inhibit the proliferation of PASMC and endothelial cells and vascular contraction by increasing I_KATP_ (Bohnen et al., 2018; Le Ribeuz et al., 2022). Activation of SUR2 has been shown in experimental models to reduce PASMC proliferation and migration. Kv1.5 is a hypoxia-sensitive Kv channel in PASMC, and its absence induces PASMC depolarization, promoting pulmonary artery contraction and remodeling. In rats with decreased Kv currents due to chronic hypoxia, upregulating the expression of the KCNA5 gene helps lower pulmonary vascular resistance and relieve PAH. These findings highlight the role of KCNA5-related potassium currents in PAH-associated vascular remodeling (Pozeg et al., 2003; Vera-Zambrano et al., 2023).

### 4.6 I/R injury

I/R injury describes the cellular dysfunction occurring after the restoration of blood supply to previously ischemic tissue. The pathological process of I/R injury is complex and diverse, making it a major cause of morbidity and mortality in CVDs (Zhang et al., 2024). Insufficient serum potassium and abnormal levels of potassium channels such as BK and K_ATP_ are involved in the pathogenesis of I/R injury.

Potassium deficiency exacerbates I/R injury by promoting cellular stress responses, including autophagy and apoptosis (Tan et al., 2019; Wang et al., 2023). K_ATP_ and BK play roles in the pathogenesis of I/R injury, but there is some controversy regarding the use of K_ATP_ and BK modulators in the treatment of I/R injury. Studies have reported divergent roles of K_ATP_ in I/R injury. Mitochondrial K_ATP_ inactivation promotes membrane depolarization and oxidative stress via enhanced NADPH oxidase activity and ROS generation, whereas pharmacological K_ATP_ inhibition may alleviate I/R injury by limiting neutrophil recruitment and inflammatory amplification (Pompermayer et al., 2007; Arni et al., 2021). BK activation significantly reduces oxidative stress by inhibiting mitochondrial ROS production, improving endocardial motion, and enhancing myocardial contractility, thus reducing I/R injury (Behmenburg et al., 2017; Goswami et al., 2018). However, overactivation of BK can increase I/R-induced damage to hippocampal neurons (Chen et al., 2013). Study indicates that in neonates, BK activation promotes apoptosis and enhances I/R-induced myocardial damage. This difference from adults may be related to changes in BK localization during cellular development (Sanghvi et al., 2022).

### 4.7 Cardiomyopathy

Cardiomyopathy refers to a group of heterogeneous myocardial diseases marked by structural and functional myocardial abnormalities (McKenna et al., 2017). Cardiomyopathies are primarily classified by phenotype into five forms: hypertrophic (HCM), dilated (DCM), restrictive (RCM), arrhythmogenic right ventricular (ARVC), and non-dilated left ventricular (NDLVC) cardiomyopathy. They can also be categorized etiologically as primary (genetic, mixed, acquired) or secondary to systemic diseases (Arbelo et al., 2023). Dysregulation of potassium homeostasis caused by the functional abnormalities of K_ATP_, NKA, and the KCNQ1 gene, contributes to the development of various cardiomyopathies.

HCM, marked by increased left ventricular wall thickness, is the most common hereditary cardiomyopathy in clinical practice (Maron and Maron, 2013). Energy metabolism imbalance is the fundamental cause of its occurrence. When K_ATP_ structural or functional disorders occur, the forkhead box protein O1 (FoxO1)/peroxisome proliferator-activated receptor gamma coactivator-1α signaling pathway related to regulating energy metabolism is inhibited, accelerating the progression of HCM (Hu et al., 2008). In a computational electrophysiological model study, HCM exhibited prolonged APD in response to β-adrenergic receptor stimulation (β-ARS), mainly due to reduced potassium repolarization current under β-ARS (Doste et al., 2022). In summary, potassium currents are vital for maintaining the electrophysiological stability and energy metabolism in HCM.

Dilated cardiomyopathy is characterized by left ventricular or biventricular dilation or systolic dysfunction, and is not associated with abnormal cardiac loading or severe coronary artery disease (Heymans et al., 2023). Studies have found that patients with dilated cardiomyopathy often have mutations in the KCNQ1 gene and the ABCC9 gene, which encodes the K_ATP_ subunit SUR2A. These findings suggest that mutations in both the KCNQ1 and K_ATP_ genes may heighten susceptibility to dilated cardiomyopathy (Bienengraeber et al., 2004; Xiong et al., 2015).

Diabetic cardiomyopathy often occurs secondary to diabetes, initially presenting as diastolic dysfunction and often progressing to HF (Jia et al., 2018). Abnormal lipid metabolism tends to speed up the progression of diabetic cardiomyopathy. Kir6.1 can accelerate lipid metabolism by activating protein kinase B and promoting the phosphorylation of FoxO1. Knockout of Kir6.1 downregulates the AKT-FoxO1 signaling pathway, exacerbating cardiac dysfunction in diabetic mice (Wang et al., 2021).

ICM features significant vascular stenosis and commonly manifests as left ventricular dysfunction (Pastena et al., 2024). Research indicates that K_ATP_ activation can decrease the production of inflammatory factors like TNF-α by inhibiting the synthesis of downstream MAPK, thus providing therapeutic benefits for ICM. Hence, K_ATP_ can affect ICM progression by intervening in inflammatory responses (**Table 11**) (Liu et al., 2016).

**Table 11:** Cardiovascular diseases associated with potassium imbalance.

Clinical Classification	Disease	Potassium Imbalance Type
Arrhythmias	AF	Hypokalemia; Potassium channel dysfunction
	PVCs	Hypokalemia; Potassium channel dysfunction
	SB	Potassium channel dysfunction
	VT	Potassium channel dysfunction; Potassium imbalance
	VF	Potassium channel dysfunction; Potassium imbalance
	LQTS	Hypokalemia; Potassium channel dysfunction
	SQTS	Potassium channel dysfunction
Non-arrhythmias	HF	Hypokalemia; Hyperkalemia; Potassium channel dysfunction
	HT	Low potassium intake; Hyperkalemia; Potassium channel dysfunction
	Atherosclerosis	Low dietary potassium; Potassium channel dysfunction
	PAH	Potassium channel dysfunction
	I/R injury	Hypokalemia; Potassium channel dysfunction
	HCM	Potassium channel dysfunction
	DCM	Potassium channel dysfunction
	ICM	Potassium channel dysfunction

AF, atrial fibrillation; PVCs, premature ventricular contractions; SB, sinus bradycardia; VT, ventricular tachycardia; VF, ventricular fibrillation; LQTS, long QT syndrome; SQTS, short QT syndrome; HF, heart failure; HT, hypertension; PAH, pulmonary arterial hypertension; I/R injury, ischemia/reperfusion injury; HCM, hypertrophic cardiomyopathy; DCM, dilated cardiomyopathy; ICM, ischemic cardiomyopathy.

## 5 Pharmacological strategies targeting potassium balance

Potassium homeostasis is fundamental to cardiovascular physiology, and its disruption is a contributor to arrhythmias, HF, and cardiomyopathy. Consequently, elucidating its regulatory mechanisms is central to understanding CVD pathogenesis. This understanding is driving the exploration of therapeutic strategies that target potassium homeostasis.

Promising experimental evidence suggests that certain natural compounds, such as curcumin (Liu et al., 2006; Liu et al., 2014; Aréchiga-Figueroa et al., 2015; Chen et al., 2015) (modulating K_ATP_, Kv, and hERG channels), ginsenoside Re (Bai et al., 2004; Sukrittanon et al., 2014), (activating SK and Ks channels), and astragaloside (Liu et al., 2018) (possibly via HCN4) can influence potassium channel activity. However, their translational potential requires rigorous clinical validation. In contrast, clinically established therapies are already shaping management. Novel potassium binders, such as patiromer and sodium zirconium cyclosilicate, offer effective and safe hyperkalemia control, enabling optimal renin-angiotensin-aldosterone system inhibitor therapy in HF and chronic kidney disease (McArthur et al., 2025; Shimada et al., 2026). Antiarrhythmic drugs like amiodarone, though indirectly affecting potassium homeostasis, remain cornerstones for rhythm control (Gelman et al., 2024). Meanwhile, more targeted agents, such as the K_Ca_ channel activator NS309, represent an emerging direction for pharmacotherapy (Kroigaard et al., 2012). Beyond pharmacology, genetic insights are revealing the roots of dyshomeostasis. Advances in genetic engineering now allow for precise modulation of channel expression, paving the way for gene-based interventions (Guo et al., 2026). Coupled with progress in multi-omics and electrophysiological techniques, these approaches foster hope for innovative, personalized treatment strategies aimed at improving CVDs outcomes (Sahu et al., 2025).

Despite significant progress, major gaps remain in understanding the integrated regulation of potassium channels, their subtype-specific interactions, and their precise roles in CVDs. Advances in electrophysiological techniques and multi-omics approaches may facilitate more targeted and translational investigations in the future.

## 6 Conclusion

In conclusion, potassium homeostasis serves as a fundamental physiological basis for maintaining cardiovascular health. This review delineates the intricate regulatory network of K^+^ within the organism. Dysregulation of this complex system, whether manifesting as hyperkalemia or hypokalemia, can disrupt the electrical stability of cardiomyocytes and significantly increase the risk of developing cardiovascular diseases, including arrhythmias, HF, atherosclerosis, and hypertension. Advancing individualized prevention and treatment strategies centered on potassium homeostasis management will therefore contribute to preserving systemic potassium homeostasis and safeguarding cardiovascular health.

## Glossary

(P)RR: the (pro)renin receptor
AERP: atrial effective refractory period
AF: atrial fibrillation
Ang II: angiotensin II
AP: action potential
APD: action potential duration
ASDN: aldosterone-sensitive distal nephron
BK: big conductance calcium-activated potassium channel
CaMKII: calmodulin-dependent protein kinase II
CCD: cortical collecting duct
CNT: connecting tubule
DCT: distal convoluted tubule
EAD: early afterdepolarization
ENaC: epithelial Na^+^ channel
ERK: extracellular signal-regulated kinase
ERP: effective refractory period
FoxO1: forkhead box protein O1
HCM: hypertrophic cardiomyopathy
HCN4: hyperpolarization-activated cyclic nucleotide-gated 4
hERG: human ether-a-go-go-related gene
HF: heart failure
I/R: ischemia-reperfusion
ICM: ischemic cardiomyopathy
I_f_: the funny current
I_K_: intermediate conductance calcium-activated potassium channel
I_K1_: inward rectifier potassium current
I_KATP_: ATP-sensitive potassium current
I_Kr_: fast delayed rectifier potassium current
I_Ks_: slow delayed rectifier potassium current
I_to_: transient outward potassium current
I_SK_: SK current
K1: inward rectifier potassium channel
K_2_P: two-pore-domain potassium channel
K_ACh_: acetylcholine-sensitive potassium channel
K_ATP_: ATP-sensitive potassium channel
K_Ca_: calcium-activated potassium channel
Kir: inwardly rectifying potassium channel
Kr: fast delayed rectifier potassium channel
Ks: slow delayed rectifier potassium channel
Kto: transient outward potassium channel
Kur: ultra-rapid delayed rectifier potassium channel
Kv: voltage-gated potassium channel
K^+^: Potassium
LQT 1: Long QT Syndrome type 1
LQT 2: Long QT Syndrome type 2
LQT 7: Long QT Syndrome type 7
LQTS: Long QT Syndrome
MAPK: mitogen-activated protein kinase
minK: minimal K^+^ channel
mTOR: mechanistic target of rapamycin
NCC: sodium-chloride cotransporter
NF-κB: nuclear factor kappa B
NKA: Na^+^-K^+^-ATPase
NKCC1: Na^+^-K^+^-2Cl^-^ cotransporter 1
OSR1: odd-skipped related 1
ox-LDL: oxidized-low density lipoprotein
PAH: pulmonary arterial hypertension
PASMC: pulmonary arterial smooth muscle cell
PP1A: phosphatase 1, α
PVC: premature ventricular contraction
RAAS: renin-angiotensin-aldosterone system
RAASi: RAAS inhibitors
RCM: restrictive cardiomyopathy
ROMK: renal outer medullary K^+^ channel
SAN: sinoatrial node
SK: small conductance calcium-activated potassium channel
SQTS: short QT Syndrome
SUR: sulfonylurea receptor
TdP: torsades de pointes
TRPV4: transient receptor potential vanilloid type 4
UCM: uremic cardiomyopathy
VF: ventricular fibrillation
VSMC: vascular smooth muscle cell
VT: ventricular tachycardia
WNK/SPAK: with no (K) lysine kinase/STE20/SPS1-related proline-alanine-rich protein kinase

## References

[B1] Abate V. Vergatti A. Altavilla N. Garofano F. Salcuni A. S. Rendina D. . (2024). Potassium intake and bone health: A narrative review. Nutrients 16 (17), 3016. doi: 10.3390/nu16173016, PMID: 39275337 PMC11397259

[B2] Agarwal R. Afzalpurkar R. Fordtran J. S. (1994). Pathophysiology of potassium absorption and secretion by the human intestine. Gastroenterology 107, 548–571. doi: 10.1016/0016-5085(94)90184-8, PMID: 8039632

[B3] AlMahameed S. T. Ziv O. (2019). Ventricular arrhythmias. Med. Clin. North Am. 103 (5), 881–95. doi: 10.1016/j.mcna.2019.05.008, PMID: 31378332

[B4] Amini M. Zayeri F. Salehi M. (2021). Trend analysis of cardiovascular disease mortality, incidence, and mortality-to-incidence ratio: results from global burden of disease study 2017. BMC Public Health 21 (1), 401. doi: 10.1186/s12889-021-10429-0, PMID: 33632204 PMC7905904

[B5] Anderson M. E. Brown J. H. Bers D. M. (2011). CaMKII in myocardial hypertrophy and heart failure. J. Mol. Cell Cardiol. 51 (4), 468–73. doi: 10.1016/j.yjmcc.2011.01.012, PMID: 21276796 PMC3158288

[B6] Antigny F. Hautefort A. Meloche J. Belacel-Ouari M. Manoury B. Rucker-Martin C. . (2016). Potassium channel subfamily K member 3 (KCNK3) contributes to the development of pulmonary arterial hypertension. Circulation 133 (14), 1371–85. doi: 10.1161/CIRCULATIONAHA.115.020951, PMID: 26912814

[B7] Arbelo E. Protonotarios A. Gimeno J. R. Arbustini E. Barriales-Villa R. Basso C. . (2023). 2023 ESC Guidelines for the management of cardiomyopathies. Eur. Heart J. 44 (37), 3503–626. doi: 10.1093/eurheartj/ehad194, PMID: 37622657

[B8] Aréchiga-Figueroa I. A. Delgado-Ramírez M. Morán-Zendejas R. Rodríguez-Menchaca A. A. (2015). Modulation of Kv2.1 channels inactivation by curcumin. Pharmacol. Reports: PR 67 (6), 1273–79. doi: 10.1016/j.pharep.2015.05.019, PMID: 26481552

[B9] Arni S. Maeyashiki T. Latshang T. Opitz I. Inci I. (2021). Ex vivo lung perfusion with K(ATP) channel modulators antagonize ischemia reperfusion injury. Cells 10 (9), 2296. doi: 10.3390/cells10092296, PMID: 34571948 PMC8472464

[B10] Aziz Q. Li Y. Anderson N. Ojake L. Tsisanova E. Tinker A. (2017). Molecular and functional characterization of the endothelial ATP-sensitive potassium channel. J. Biol. Chem. 292 (43), 17587–97. doi: 10.1074/jbc.M117.810325, PMID: 28893911 PMC5663864

[B11] Bai C. X. Takahashi K. Masumiya H. Sawanobori T. Furukawa T. (2004). Nitric oxide-dependent modulation of the delayed rectifier K+ current and the L-type Ca2+ current by ginsenoside Re, an ingredient of Panax ginseng, in Guinea-pig cardiomyocytes. Br. J. Pharmacol. 142 (3), 567–75. doi: 10.1038/sj.bjp.0705814, PMID: 15148247 PMC1574975

[B12] Bailey R. L. Ard J. D. Davis T. A. Naimi T. S. Schneeman B. O. Stang J. S. . (2021). A proposed framework for identifying nutrients and food components of public health relevance in the dietary guidelines for americans. J. Nutr. 151 (5), 1197–204. doi: 10.1093/jn/nxaa459, PMID: 33693925 PMC8324230

[B13] Baman J. R. Ahmad F. S. (2020). Heart failure. Jama 324 (10), 1015. doi: 10.1001/jama.2020.13310, PMID: 32749448

[B14] Behmenburg F. Hölscher N. Flögel U. Hollmann M. W. Heinen A. Huhn R. (2017). Opening of calcium-activated potassium channels improves long-term left-ventricular function after coronary artery occlusion in mice. Int. J. Cardiol. 241, 351–57. doi: 10.1016/j.ijcard.2017.04.084, PMID: 28487150

[B15] Bichet D. Haass F. A. Jan L. Y. (2003). Merging functional studies with structures of inward-rectifier K(+) channels. Nat. Rev. Neurosci. 4 (12), 957–67. doi: 10.1038/nrn1244, PMID: 14618155

[B16] Bidaud I. D’Souza A. Forte G. Torre E. Greuet D. Thirard S. . (2020). Genetic ablation of G protein-gated inwardly rectifying K(+) channels prevents training-induced sinus bradycardia. Front. Physiol. 11, 519382. doi: 10.3389/fphys.2020.519382, PMID: 33551824 PMC7857143

[B17] Bienengraeber M. Olson T. M. Selivanov V. A. Kathmann E. C. O’Cochlain F. Gao F. . (2004). ABCC9 mutations identified in human dilated cardiomyopathy disrupt catalytic KATP channel gating. Nat. Genet. 36 (4), 382–87. doi: 10.1038/ng1329, PMID: 15034580 PMC1995438

[B18] Binaghi G. Abrignani M. G. Temporelli P. L. Casula M. Maloberti A. Riccio C. . (2025). Potassium role in the human body and clinical implications of hyperkalemia. Giornale Italiano Di Cardiol. (2006) 26 (1), 50–60. doi: 10.1714/4394.43959, PMID: 39714500

[B19] Bjerregaard P. (2018). Diagnosis and management of short QT syndrome. Heart Rhythm. 15 (8), 1261–67. doi: 10.1016/j.hrthm.2018.02.034, PMID: 29501667

[B20] Blanchard A. Bockenhauer D. Bolignano D. Calò L. A. Cosyns E. Devuyst O. . (2017). Gitelman syndrome: consensus and guidance from a Kidney Disease: Improving Global Outcomes (KDIGO) Controversies Conference. Kidney Int. 91 (1), 24–33. doi: 10.1016/j.kint.2016.09.046, PMID: 28003083

[B21] Bohnen M. S. Ma L. Zhu N. Qi H. McClenaghan C. Gonzaga-Jauregui C. . (2018). Loss-of-function ABCC8 mutations in pulmonary arterial hypertension. Circ. Genom. Precis. Med. 11 (10), e002087. doi: 10.1161/CIRCGEN.118.002087, PMID: 30354297 PMC6206877

[B22] Brähler S. Kaistha A. Schmidt V. J. Wölfle S. E. Busch C. Kaistha B. P. . (2009). Genetic deficit of SK3 and IK1 channels disrupts the endothelium-derived hyperpolarizing factor vasodilator pathway and causes hypertension. Circulation 119 (17), 2323–32. doi: 10.1161/CIRCULATIONAHA.108.846634, PMID: 19380617

[B23] Brown B. M. Shim H. Christophersen P. Wulff H. (2020). Pharmacology of small- and intermediate-conductance calcium-activated potassium channels. Annu. Rev. Pharmacol. Toxicol. 60, 219–40. doi: 10.1146/annurev-pharmtox-010919-023420, PMID: 31337271 PMC9615427

[B24] Brundel B. Ai X. Hills M. T. Kuipers M. F. Lip G. Y. H. de Groot N. M. S. (2022). Atrial fibrillation. Nat. Rev. Dis. Primers. 8 (1), 21. doi: 10.1038/s41572-022-00347-9, PMID: 35393446

[B25] Burg S. Attali B. (2021). Targeting of potassium channels in cardiac arrhythmias. Trends Pharmacol. Sci. 42 (6), 491–506. doi: 10.1016/j.tips.2021.03.005, PMID: 33858691

[B26] Burnier M. Damianaki A. (2023). Hypertension as cardiovascular risk factor in chronic kidney disease. Circ. Res. 132 (8), 1050–63. doi: 10.1161/CIRCRESAHA.122.321762, PMID: 37053276

[B27] Byrd J. B. Brook R. D. (2019). Hypertension. Ann. Intern. Med. 170 (9), ITC65–80. doi: 10.7326/AITC201905070, PMID: 31060074

[B28] Caravaca Perez P. González-Juanatey J. R. Nuche J. Morán Fernández L. Lora Pablos D. Alvarez-García J. . (2022). Serum potassium dynamics during acute heart failure hospitalization. Clin. Res. Cardiol. 111 (4), 368–79. doi: 10.1007/s00392-020-01753-3, PMID: 33070219

[B29] Castañeda-Bueno M. Cervantes-Perez L. G. Rojas-Vega L. Arroyo-Garza I. Vázquez N. Moreno E. . (2014). Modulation of NCC activity by low and high K(+) intake: insights into the signaling pathways involved. Am. J. Physiol. Renal Physiol. 306 (12), F1507–19. doi: 10.1152/ajprenal.00255.2013, PMID: 24761002 PMC4059971

[B30] Castañeda-Bueno M. Ellison D. H. Gamba G. (2022). Molecular mechanisms for the modulation of blood pressure and potassium homeostasis by the distal convoluted tubule. EMBO Mol. Med. 14 (1), e14273. doi: 10.15252/emmm.202114273, PMID: 34927382 PMC8819348

[B31] Castro H. Raij L. (2013). Potassium in hypertension and cardiovascular disease. Semin. Nephrol. 33 (3), 277–89. doi: 10.1016/j.semnephrol.2013.04.008, PMID: 23953805

[B32] Chang P. C. Chen P. S. (2015). SK channels and ventricular arrhythmias in heart failure. Trends Cardiovasc. Med. 25 (6), 508–14. doi: 10.1016/j.tcm.2015.01.010, PMID: 25743622 PMC4519433

[B33] Chen L. Sampson K. J. Kass R. S. (2016). Cardiac delayed rectifier potassium channels in health and disease. Card Electrophysiol. Clin. 8 (2), 307–22. doi: 10.1016/j.ccep.2016.01.004, PMID: 27261823 PMC4893812

[B34] Chen M. Sun H. Y. Hu P. Wang C. F. Li B. X. Li S. J. . (2013). Activation of BKca channels mediates hippocampal neuronal death after reoxygenation and reperfusion. Mol. Neurobiol. 48 (3), 794–807. doi: 10.1007/s12035-013-8467-x, PMID: 23653329

[B35] Chen Q. Tao J. Hei H. Li F. Wang Y. Peng W. . (2015). Up-regulatory effects of curcumin on large conductance ca2+-activated K+ Channels. PloS One 10, e0144800. doi: 10.1371/journal.pone.0144800, PMID: 26672753 PMC4682634

[B36] Chun T. U. Epstein M. R. Dick M. Andelfinger G. Ballester L. Vanoye C. G. . (2004). Polymorphic ventricular tachycardia and KCNJ2 mutations. Heart Rhythm 1, 235–241. doi: 10.1016/j.hrthm.2004.02.017, PMID: 15851159

[B37] Cogswell M. E. Zhang Z. Carriquiry A. L. Gunn J. P. Kuklina E. V. Saydah S. H. . (2012). Sodium and potassium intakes among US adults: NHANES 2003-2008. Am. J. Clin. Nutr. 96, 647–657. doi: 10.3945/ajcn.112.034413, PMID: 22854410 PMC3417219

[B38] Cook E. E. Davis J. Israni R. Mu F. Betts K. A. Anzalone D. . (2021). Prevalence of metabolic acidosis among patients with chronic kidney disease and hyperkalemia. Adv. Ther. 38, 5238–5252. doi: 10.1007/s12325-021-01886-5, PMID: 34471991 PMC8478736

[B39] Czech A. Zarycka E. Yanovych D. Zasadna Z. Grzegorczyk I. Kłys S. (2020). Mineral content of the pulp and peel of various citrus fruit cultivars. Biol. Trace Elem. Res. 193, 555–563. doi: 10.1007/s12011-019-01727-1, PMID: 31030384 PMC6944645

[B40] D’Elia L. Cappuccio F. P. Masulli M. La Fata E. Rendina D. Galletti F. (2023). Effect of potassium supplementation on endothelial function: A systematic review and meta-analysis of intervention studies. Nutrients 15 (4), 853. doi: 10.3390/nu15040853, PMID: 36839211 PMC9961878

[B41] Das U. N. (2002). Is insulin an endogenous cardioprotector? Crit. Care 6, 389–393. doi: 10.1186/cc1535, PMID: 12398773 PMC137317

[B42] DeFronzo R. A. Bia M. Birkhead G. (1981). Epinephrine and potassium homeostasis. Kidney Int. 20, 83–91. doi: 10.1038/ki.1981.108, PMID: 7300118

[B43] Doste R. Coppini R. Bueno-Orovio A. (2022). Remodelling of potassium currents underlies arrhythmic action potential prolongation under beta-adrenergic stimulation in hypertrophic cardiomyopathy. J. Mol. Cell Cardiol. 172, 120–131. doi: 10.1016/j.yjmcc.2022.08.361, PMID: 36058298

[B44] DuBose T. D. Jr. (2017). Regulation of potassium homeostasis in CKD. Adv. Chronic Kidney Dis. 24, 305–314. doi: 10.1053/j.ackd.2017.06.002, PMID: 29031357

[B45] Ekmekcioglu C. Elmadfa I. Meyer A. L. Moeslinger T. (2016). The role of dietary potassium in hypertension and diabetes. J. Physiol. Biochem. 72, 93–106. doi: 10.1007/s13105-015-0449-1, PMID: 26634368

[B46] Fan Y. Wu M. Li X. Zhao J. Shi J. Ding L. . (2024). Potassium levels and the risk of all-cause and cardiovascular mortality among patients with cardiovascular diseases: a meta-analysis of cohort studies. Nutr. J. 23, 8. doi: 10.1186/s12937-023-00888-z, PMID: 38195532 PMC10777575

[B47] Farah R. Nassar M. Aboraya B. Nseir W. (2021). Low serum potassium levels are associated with the risk of atrial fibrillation. Acta Cardiol. 76, 887–890. doi: 10.1080/00015385.2020.1799573, PMID: 32723154

[B48] Feghaly J. Zakka P. London B. MacRae C. A. Refaat M. M. (2018). Genetics of atrial fibrillation. J. Am. Heart Assoc. 7, e009884. doi: 10.1161/JAHA.118.009884, PMID: 30371258 PMC6474960

[B49] Foster M. N. Coetzee W. A. (2016). KATP channels in the cardiovascular system. Physiol. Rev. 96, 177–252. doi: 10.1152/physrev.00003.2015, PMID: 26660852 PMC4698399

[B50] Galiè N. Humbert M. Vachiery J. L. Gibbs S. Lang I. Torbicki A. . (2016). 2015 ESC/ERS Guidelines for the diagnosis and treatment of pulmonary hypertension: The Joint Task Force for the Diagnosis and Treatment of Pulmonary Hypertension of the European Society of Cardiology (ESC) and the European Respiratory Society (ERS): Endorsed by: Association for European Paediatric and Congenital Cardiology (AEPC), International Society for Heart and Lung Transplantation (ISHLT). Eur. Heart J. 37, 67–119. doi: 10.1093/eurheartj/ehv317, PMID: 26320113

[B51] Gamba G. (2023). Thirty years of the NaCl cotransporter: from cloning to physiology and structure. Am. J. Physiol. Renal Physiol. 325, F479–Ff90. doi: 10.1152/ajprenal.00114.2023, PMID: 37560773 PMC10639029

[B52] Gamba G. Ellison D. H. (2025). KS-WNK1 augments the effects of dietary potassium intake on renal sodium chloride reabsorption. J. Clin. Invest. 135 (15), e195512. doi: 10.1172/JCI195512, PMID: 40759567 PMC12321400

[B53] Gao P. (2019). Recent cardiovascular research highlights from China. Cardiovasc. Res. 115, e37–ee8. doi: 10.1093/cvr/cvy245, PMID: 30789232

[B54] Gao Z. Sierra A. Zhu Z. Koganti S. R. Subbotina E. Maheshwari A. . (2016). Loss of ATP-sensitive potassium channel surface expression in heart failure underlies dysregulation of action potential duration and myocardial vulnerability to injury. PloS One 11, e0151337. doi: 10.1371/journal.pone.0151337, PMID: 26964104 PMC4786327

[B55] Gelman I. Sharma N. McKeeman O. Lee P. Campagna N. Tomei N. . (2024). The ion channel basis of pharmacological effects of amiodarone on myocardial electrophysiological properties, a comprehensive review. Biomed. Pharmacother. Biomed. Pharmacother. 174, 116513. doi: 10.1016/j.biopha.2024.116513, PMID: 38565056

[B56] Goswami S. K. Ponnalagu D. Hussain A. T. Shah K. Karekar P. Gururaja Rao S. . (2018). Expression and activation of BK(Ca) channels in mice protects against ischemia-reperfusion injury of isolated hearts by modulating mitochondrial function. Front. Cardiovasc. Med. 5, 194. doi: 10.3389/fcvm.2018.00194, PMID: 30746365 PMC6360169

[B57] Greenlee M. Wingo C. S. McDonough A. A. Youn J. H. Kone B. C. (2009). Narrative review: evolving concepts in potassium homeostasis and hypokalemia. Ann. Intern. Med. 150, 619–625. doi: 10.7326/0003-4819-150-9-200905050-00008, PMID: 19414841 PMC4944758

[B58] Grimm P. R. Tatomir A. Rosenbaek L. L. Kim B. Y. Li D. Delpire E. J. . (2023). Dietary potassium stimulates Ppp1Ca-Ppp1r1a dephosphorylation of kidney NaCl cotransporter and reduces blood pressure. J. Clin. Invest. 133 (21), e158498. doi: 10.1172/JCI158498, PMID: 37676724 PMC10617769

[B59] Gumz M. L. Rabinowitz L. (2013). Role of circadian rhythms in potassium homeostasis. Semin. Nephrol. 33, 229–236. doi: 10.1016/j.semnephrol.2013.04.003, PMID: 23953800 PMC3803104

[B60] Guo J. Yang Z. Zhang X. Liu F. Ma M. Yu F. . (2026). Structure of Chlamydomonas reinhardtii LciA guided the engineering of FNT family proteins to gain bicarbonate transport activity. Nat. Plants 12, 231–240. doi: 10.1038/s41477-025-02200-9, PMID: 41507353

[B61] Haddy F. J. Vanhoutte P. M. Feletou M. (2006). Role of potassium in regulating blood flow and blood pressure. Am. J. Physiol. Regul. Integr. Comp. Physiol. 290, R546–R552. doi: 10.1152/ajpregu.00491.2005, PMID: 16467502

[B62] Hancox J. C. Du C. Y. Butler A. Zhang Y. Dempsey C. E. Harmer S. C. . (2023). Pro-arrhythmic effects of gain-of-function potassium channel mutations in the short QT syndrome. Philos. Trans. R. Soc. Lond. B. Biol. Sci. 378, 20220165. doi: 10.1098/rstb.2022.0165, PMID: 37122211 PMC10150212

[B63] He Q. Feng Y. Wang Y. (2015). Transient outward potassium channel: a heart failure mediator. Heart Fail Rev. 20, 349–362. doi: 10.1007/s10741-015-9474-y, PMID: 25646587

[B64] He F. J. MacGregor G. A. (2008). Beneficial effects of potassium on human health. Physiol. Plant 133, 725–735. doi: 10.1111/j.1399-3054.2007.01033.x, PMID: 18724413

[B65] Heijman J. Zhou X. Morotti S. Molina C. E. Abu-Taha I. H. Tekook M. . (2023). Enhanced ca(2+)-dependent SK-channel gating and membrane trafficking in human atrial fibrillation. Circ. Res. 132, e116–ee33. doi: 10.1161/CIRCRESAHA.122.321858, PMID: 36927079 PMC10147588

[B66] Heymans S. Lakdawala N. K. Tschöpe C. Klingel K. (2023). Dilated cardiomyopathy: causes, mechanisms, and current and future treatment approaches. Lancet 402, 998–1011. doi: 10.1016/S0140-6736(23)01241-2, PMID: 37716772

[B67] Hibino H. Inanobe A. Furutani K. Murakami S. Findlay I. Kurachi Y. (2010). Inwardly rectifying potassium channels: their structure, function, and physiological roles. Physiol. Rev. 90, 291–366. doi: 10.1152/physrev.00021.2009, PMID: 20086079

[B68] Hoogendijk M. G. Géczy T. Yap S. C. Szili-Torok T. (2020). Pathophysiological mechanisms of premature ventricular complexes. Front. Physiol. 11, 406. doi: 10.3389/fphys.2020.00406, PMID: 32528299 PMC7247859

[B69] Hu X. Xu X. Huang Y. Fassett J. Flagg T. P. Zhang Y. . (2008). Disruption of sarcolemmal ATP-sensitive potassium channel activity impairs the cardiac response to systolic overload. Circ. Res. 103, 1009–1017. doi: 10.1161/CIRCRESAHA.107.170795, PMID: 18802029 PMC2877276

[B70] Husti Z. Varró A. Baczkó I. (2021). Arrhythmogenic remodeling in the failing heart. Cells 10 (11), 3203. doi: 10.3390/cells10113203, PMID: 34831426 PMC8623396

[B71] Jalife J. (2009). Inward rectifier potassium channels control rotor frequency in ventricular fibrillation. Heart Rhythm. 6, S44–S48. doi: 10.1016/j.hrthm.2009.07.019, PMID: 19880073 PMC2782423

[B72] Jia G. Whaley-Connell A. Sowers J. R. (2018). Diabetic cardiomyopathy: a hyperglycaemia- and insulin-resistance-induced heart disease. Diabetologia 61, 21–28. doi: 10.1007/s00125-017-4390-4, PMID: 28776083 PMC5720913

[B73] Jiang X. X. Bian W. Zhu Y. R. Wang Z. Ye P. Gu Y. . (2022). Targeting the KCa3.1 channel suppresses diabetes-associated atherosclerosis via the STAT3/CD36 axis. Diabetes Res. Clin. Pract. 185, 109776. doi: 10.1016/j.diabres.2022.109776, PMID: 35149165

[B74] Kan X. H. Gao H. Q. Ma Z. Y. Liu L. Ling M. Y. Wang Y. Y. (2016). Kv1.3 potassium channel mediates macrophage migration in atherosclerosis by regulating ERK activity. Arch. Biochem. Biophys. 591, 150–156. doi: 10.1016/j.abb.2015.12.013, PMID: 26748289

[B75] Kellum J. A. (2005). Clinical review: reunification of acid-base physiology. Crit. Care 9, 500–507. doi: 10.1186/cc3789, PMID: 16277739 PMC1297616

[B76] Kettritz R. Loffing J. (2023). Potassium homeostasis - Physiology and pharmacology in a clinical context. Pharmacol. Ther. 249, 108489. doi: 10.1016/j.pharmthera.2023.108489, PMID: 37454737

[B77] Kim M. J. Valerio C. Knobloch G. K. (2023). Potassium disorders: hypokalemia and hyperkalemia. Am. Fam. Physician. 107, 59–70. 36689973

[B78] Kovesdy C. P. Appel L. J. Grams M. E. Gutekunst L. McCullough P. A. Palmer B. F. . (2017). Potassium homeostasis in health and disease: A scientific workshop cosponsored by the national kidney foundation and the american society of hypertension. Am. J. Kidney Dis. 70, 844–858. doi: 10.1053/j.ajkd.2017.09.003, PMID: 29029808

[B79] Krahn A. D. Laksman Z. Sy R. W. Postema P. G. Ackerman M. J. Wilde A. A. M. . (2022). Congenital long QT syndrome. JACC Clin. Electrophysiol. 8, 687–706. doi: 10.1016/j.jacep.2022.02.017, PMID: 35589186

[B80] Krapivinsky G. Gordon E. A. Wickman K. Velimirović B. Krapivinsky L. Clapham D. E. (1995). The G-protein-gated atrial K+ channel IKACh is a heteromultimer of two inwardly rectifying K(+)-channel proteins. Nature 374, 135–141. doi: 10.1038/374135a0, PMID: 7877685

[B81] Krogager M. L. Kragholm K. Thomassen J. Q. Søgaard P. Lewis B. S. Wassmann S. . (2021). Update on management of hypokalaemia and goals for the lower potassium level in patients with cardiovascular disease: a review in collaboration with the European Society of Cardiology Working Group on Cardiovascular Pharmacotherapy. Eur. Heart J. Cardiovasc. Pharmacother. 7, 557–567. doi: 10.1093/ehjcvp/pvab038, PMID: 33956964

[B82] Kroigaard C. Dalsgaard T. Nielsen G. Laursen B. E. Pilegaard H. Köhler R. . (2012). Activation of endothelial and epithelial K(Ca) 2.3 calcium-activated potassium channels by NS309 relaxes human small pulmonary arteries and bronchioles. Br. J. Pharmacol. 167, 37–47. doi: 10.1111/j.1476-5381.2012.01986.x, PMID: 22506557 PMC3448912

[B83] Kusumoto F. M. Schoenfeld M. H. Barrett C. Edgerton J. R. Ellenbogen K. A. Gold M. R. . (2019). 2018 ACC/AHA/HRS guideline on the evaluation and management of patients with bradycardia and cardiac conduction delay: A report of the american college of cardiology/american heart association task force on clinical practice guidelines and the heart rhythm society. Circulation 140, e382–e482. doi: 10.1161/CIR.0000000000000628, PMID: 30586772

[B84] Lambert M. Boet A. Rucker-Martin C. Mendes-Ferreira P. Capuano V. Hatem S. . (2018). Loss of KCNK3 is a hallmark of RV hypertrophy/dysfunction associated with pulmonary hypertension. Cardiovasc. Res. 114, 880–893. doi: 10.1093/cvr/cvy016, PMID: 29360952

[B85] Larivée N. L. Michaud J. B. More K. M. Wilson J. A. Tennankore K. K. (2023). Hyperkalemia: prevalence, predictors and emerging treatments. Cardiol. Ther. 12, 35–63. doi: 10.1007/s40119-022-00289-z, PMID: 36503972 PMC9742042

[B86] Latorre R. Castillo K. Carrasquel-Ursulaez W. Sepulveda R. V. Gonzalez-Nilo F. Gonzalez C. . (2017). Molecular determinants of BK channel functional diversity and functioning. Physiol. Rev. 97, 39–87. doi: 10.1152/physrev.00001.2016, PMID: 27807200

[B87] Lees-Miller J. P. Guo J. Somers J. R. Roach D. E. Sheldon R. S. Rancourt D. E. . (2003). Selective knockout of mouse ERG1 B potassium channel eliminates I(Kr) in adult ventricular myocytes and elicits episodes of abrupt sinus bradycardia. Mol. Cell Biol. 23, 1856–1862. doi: 10.1128/MCB.23.6.1856-1862.2003, PMID: 12612061 PMC149456

[B88] Le Ribeuz H. Masson B. Dutheil M. Boët A. Beauvais A. Sabourin J. . (2022). Involvement of SUR2/Kir6.1 channel in the physiopathology of pulmonary arterial hypertension. Front. Cardiovasc. Med. 9, 1066047. doi: 10.3389/fcvm.2022.1066047, PMID: 36704469 PMC9871631

[B89] Li Y. Aziz Q. Anderson N. Ojake L. Tinker A. (2020). Endothelial ATP-sensitive potassium channel protects against the development of hypertension and atherosclerosis. Hypertension 76, 776–784. doi: 10.1161/HYPERTENSIONAHA.120.15355, PMID: 32654556 PMC7418932

[B90] Lindinger M. I. Cairns S. P. (2021). Regulation of muscle potassium: exercise performance, fatigue and health implications. Eur. J. Appl. Physiol. 121, 721–748. doi: 10.1007/s00421-020-04546-8, PMID: 33392745

[B91] Ling M. Y. Ma Z. Y. Wang Y. Y. Qi J. Liu L. Li L. . (2013). Up-regulated ATP-sensitive potassium channels play a role in increased inflammation and plaque vulnerability in macrophages. Atherosclerosis 226, 348–355. doi: 10.1016/j.atherosclerosis.2012.11.016, PMID: 23218803

[B92] Little R. Murali S. K. Poulsen S. B. Grimm P. R. Assmus A. Cheng L. . (2023). Dissociation of sodium-chloride cotransporter expression and blood pressure during chronic high dietary potassium supplementation. JCI Insight 8 (5), e156437. doi: 10.1172/jci.insight.156437, PMID: 36719746 PMC10077486

[B93] Liu Z. Cai H. Dang Y. Qiu C. Wang J. (2016). Adenosine triphosphate-sensitive potassium channels and cardiomyopathies (Review). Mol. Med. Rep. 13, 1447–1454. doi: 10.3892/mmr.2015.4714, PMID: 26707080

[B94] Liu H. Danthi S. J. Enyeart J. J. (2006). Curcumin potently blocks Kv1.4 potassium channels. Biochem. Biophys. Res. Commun. 344, 1161–1165. doi: 10.1016/j.bbrc.2006.04.020, PMID: 16647042 PMC2656109

[B95] Liu C. Lai Y. Pei J. Huang H. Zhan J. Ying S. . (2021). Clinical and genetic analysis of KATP variants with heart failure risk in patients with decreased serum apoA-I levels. J. Clin. Endocrinol. Metab. 106, 2264–2278. doi: 10.1210/clinem/dgab336, PMID: 33982099

[B96] Liu R. Li J. Liu Y. Peng J. Guan X. (2018). The effect of astragaloside on pacemaker current and the cytoskeleton in rabbit sinoatrial node cells under the ischemia and reperfusion condition. Front. Pharmacol. 9, 551. doi: 10.3389/fphar.2018.00551, PMID: 29899698 PMC5988886

[B97] Liu T. Li T. Xu D. Wang Y. Zhou Y. Wan J. . (2023). Small-conductance calcium-activated potassium channels in the heart: expression, regulation and pathological implications. Philos. Trans. R. Soc. Lond. B. Biol. Sci. 378, 20220171. doi: 10.1098/rstb.2022.0171, PMID: 37122223 PMC10150224

[B98] Liu Y. Song X. Shi Y. Shi Z. Niu W. Feng X. . (2015). WNK1 activates large-conductance Ca2+-activated K+ channels through modulation of ERK1/2 signaling. J. Am. Soc. Nephrol. 26, 844–854. doi: 10.1681/ASN.2014020186, PMID: 25145935 PMC4378107

[B99] Liu X. Sun K. Song A. Zhang X. Zhang X. He X. (2014). Curcumin inhibits proliferation of gastric cancer cells by impairing ATP-sensitive potassium channel opening. World J. Surg. Oncol. 12, 389. doi: 10.1186/1477-7819-12-389, PMID: 25523120 PMC4395964

[B100] Long C. L. Qin X. C. Pan Z. Y. Chen K. Zhang Y. F. Cui W. Y. . (2008). Activation of ATP-sensitive potassium channels protects vascular endothelial cells from hypertension and renal injury induced by hyperuricemia. J. Hypertens. 26, 2326–2338. doi: 10.1097/HJH.0b013e328312c8c1, PMID: 19008712

[B101] Lozano-Velasco E. Franco D. Aranega A. Daimi H. (2020). Genetics and epigenetics of atrial fibrillation. Int. J. Mol. Sci. 21 (16), 5717. doi: 10.3390/ijms21165717, PMID: 32784971 PMC7460853

[B102] Mabillard H. Sayer J. A. (2019). The molecular genetics of gordon syndrome. Genes (Basel) 10 (12), 986. doi: 10.3390/genes10120986, PMID: 31795491 PMC6947027

[B103] Macdonald J. E. Struthers A. D. (2004). What is the optimal serum potassium level in cardiovascular patients? J. Am. Coll. Cardiol. 43, 155–161. doi: 10.1016/j.jacc.2003.06.021, PMID: 14736430

[B104] MacRae C. A. Roden D. M. Loscalzo J. (2016). The future of cardiovascular therapeutics. Circulation 133, 2610–2617. doi: 10.1161/CIRCULATIONAHA.116.023555, PMID: 27324356

[B105] Mahmood S. S. Levy D. Vasan R. S. Wang T. J. (2014). The Framingham Heart Study and the epidemiology of cardiovascular disease: a historical perspective. Lancet 383, 999–1008. doi: 10.1016/S0140-6736(13)61752-3, PMID: 24084292 PMC4159698

[B106] Mamenko M. V. Boukelmoune N. Tomilin V. N. Zaika O. L. Jensen V. B. O’Neil R. G. . (2017). The renal TRPV4 channel is essential for adaptation to increased dietary potassium. Kidney Int. 91, 1398–1409. doi: 10.1016/j.kint.2016.12.010, PMID: 28187982 PMC5429991

[B107] Mantovani A. Sica A. Sozzani S. Allavena P. Vecchi A. Locati M. (2004). The chemokine system in diverse forms of macrophage activation and polarization. Trends Immunol. 25, 677–686. doi: 10.1016/j.it.2004.09.015, PMID: 15530839

[B108] Maron B. J. Maron M. S. (2013). Hypertrophic cardiomyopathy. Lancet 381, 242–255. doi: 10.1016/S0140-6736(12)60397-3, PMID: 22874472

[B109] Martin R. S. Panese S. Virginillo M. Gimenez M. Litardo M. Arrizurieta E. . (1986). Increased secretion of potassium in the rectum of humans with chronic renal failure. Am. J. Kidney Dis. 8, 105–110. doi: 10.1016/S0272-6386(86)80120-2, PMID: 3740056

[B110] McArthur R. Peacock W. F. Budden J. Rafique Z. (2025). Net clinical benefit of patiromer for acute hyperkalemia: a *post-hoc* analysis of the reduce trial. Ann. Med. 57, 2581912. doi: 10.1080/07853890.2025.2581912, PMID: 41178555 PMC12584821

[B111] McDonough A. A. Youn J. H. (2005). Role of muscle in regulating extracellular [K+. Semin. Nephrol. 25, 335–342. doi: 10.1016/j.semnephrol.2005.03.009, PMID: 16139689

[B112] McKenna W. J. Maron B. J. Thiene G. (2017). Classification, epidemiology, and global burden of cardiomyopathies. Circ. Res. 121, 722–730. doi: 10.1161/CIRCRESAHA.117.309711, PMID: 28912179

[B113] McLean R. M. Wang N. X. (2021). Potassium. Adv. Food Nutr. Res. 96, 89–121. doi: 10.1016/bs.afnr.2021.02.013, PMID: 34112360

[B114] Mutig K. Bachmann S. (2019). Hyperkalemia and blood pressure regulation. Nephrol. Dial Transpl. 34, iii26–iii35. doi: 10.1093/ndt/gfz218, PMID: 31800077

[B115] Nesterov V. Bertog M. Korbmacher C. (2022). High baseline ROMK activity in the mouse late distal convoluted and early connecting tubule probably contributes to aldosterone-independent K(+) secretion. Am. J. Physiol. Renal Physiol. 322, F42–f54. doi: 10.1152/ajprenal.00252.2021, PMID: 34843658

[B116] Nickerson A. J. Rajendran V. M. (2021). Aldosterone up-regulates basolateral Na(+) -K(+) -2Cl(-) cotransporter-1 to support enhanced large-conductance K(+) channel-mediated K(+) secretion in rat distal colon. FASEB J. 35, e21606. doi: 10.1096/fj.202100203R, PMID: 33908679 PMC9777186

[B117] Nilsson E. Gasparini A. Ärnlöv J. Xu H. Henriksson K. M. Coresh J. . (2017). Incidence and determinants of hyperkalemia and hypokalemia in a large healthcare system. Int. J. Cardiol. 245, 277–284. doi: 10.1016/j.ijcard.2017.07.035, PMID: 28735756

[B118] Nishiyama A. Kambe F. Kamiya K. Yamaguchi S. Murata Y. Seo H. . (1997). Effects of thyroid and glucocorticoid hormones on Kv1.5 potassium channel gene expression in the rat left ventricle. Biochem. Biophys. Res. Commun. 237, 521–526. doi: 10.1006/bbrc.1997.7182, PMID: 9299396

[B119] Orfali R. Albanyan N. (2023). Ca(2+)-sensitive potassium channels. Molecules 28 (2), 885. doi: 10.3390/molecules28020885, PMID: 36677942 PMC9861210

[B120] Palmer B. F. (2015). Regulation of potassium homeostasis. Clin. J. Am. Soc. Nephrol. 10, 1050–1060. doi: 10.2215/CJN.08580813, PMID: 24721891 PMC4455213

[B121] Palmer B. F. Clegg D. J. (2019). Physiology and pathophysiology of potassium homeostasis: core curriculum 2019. Am. J. Kidney Dis.: Off. J. Natl. Kidney Foundation 74, 682–695. doi: 10.1053/j.ajkd.2019.03.427, PMID: 31227226

[B122] Pastena P. Frye J. T. Ho C. Goldschmidt M. E. Kalogeropoulos A. P. (2024). Ischemic cardiomyopathy: epidemiology, pathophysiology, outcomes, and therapeutic options. Heart Fail Rev. 29, 287–299. doi: 10.1007/s10741-023-10377-4, PMID: 38103139

[B123] Perrotta I. (2023). Atherosclerosis: from molecular biology to therapeutic perspective 3.0. Int. J. Mol. Sci. 24 (8), 6897. doi: 10.3390/ijms24086897, PMID: 37108058 PMC10138640

[B124] Podrid P. J. (1990). Potassium and ventricular arrhythmias. Am. J. Cardiol. 65, 33E–44E. doi: 10.1016/0002-9149(90)90250-5, PMID: 2178376

[B125] Pompermayer K. Amaral F. A. Fagundes C. T. Vieira A. T. Cunha F. Q. Teixeira M. M. . (2007). Effects of the treatment with glibenclamide, an ATP-sensitive potassium channel blocker, on intestinal ischemia and reperfusion injury. Eur. J. Pharmacol. 556, 215–222. doi: 10.1016/j.ejphar.2006.10.065, PMID: 17182029

[B126] Pozeg Z. I. Michelakis E. D. McMurtry M. S. Thébaud B. Wu X. C. Dyck J. R. . (2003). *In vivo* gene transfer of the O2-sensitive potassium channel Kv1.5 reduces pulmonary hypertension and restores hypoxic pulmonary vasoconstriction in chronically hypoxic rats. Circulation 107, 2037–2044. doi: 10.1161/01.CIR.0000062688.76508.B3, PMID: 12695303

[B127] Rabinowitz L. (1996). Aldosterone and potassium homeostasis. Kidney Int. 49, 1738–1742. doi: 10.1038/ki.1996.258, PMID: 8743488

[B128] Rabinowitz L. Aizman R. I. (1993). The central nervous system in potassium homeostasis. Front. Neuroendocrinol. 14, 1–26. doi: 10.1006/frne.1993.1001, PMID: 8477870

[B129] Ramos C. I. González-Ortiz A. Espinosa-Cuevas A. Avesani C. M. Carrero J. J. Cuppari L. (2021). Does dietary potassium intake associate with hyperkalemia in patients with chronic kidney disease? Nephrol. Dial Transplant. 36, 2049–2057. doi: 10.1093/ndt/gfaa232, PMID: 33247727

[B130] Reilly L. Alvarado F. J. Lang D. Abozeid S. Van Ert H. Spellman C. . (2020). Genetic loss of I(K1) causes adrenergic-induced phase 3 early afterdepolariz ations and polymorphic and bidirectional ventricular tachycardia. Circ. Arrhythm Electrophysiol. 13, e008638. doi: 10.1161/CIRCEP.120.008638, PMID: 32931337 PMC7574954

[B131] Rizzoli R. (2014). Dairy products, yogurts, and bone health. Am. J. Clin. Nutr. 99, 1256s–1262s. doi: 10.3945/ajcn.113.073056, PMID: 24695889

[B132] Rodan A. R. (2017). Potassium: friend or foe? Pediatr. Nephrol. 32, 1109–1121. doi: 10.1007/s00467-016-3411-8, PMID: 27194424 PMC5115995

[B133] Rodríguez-Soriano J. (1998). Bartter and related syndromes: the puzzle is almost solved. Pediatr. Nephrol. 12, 315–327. doi: 10.1007/s004670050461, PMID: 9655365

[B134] Rosa R. M. Silva P. Young J. B. Landsberg L. Brown R. S. Rowe J. W. . (1980). Adrenergic modulation of extrarenal potassium disposal. N. Engl. J. Med. 302, 431–434. doi: 10.1056/NEJM198002213020803, PMID: 6101508

[B135] Rosano G. M. C. Tamargo J. Kjeldsen K. P. Lainscak M. Agewall S. Anker S. D. . (2018). Expert consensus document on the management of hyperkalaemia in patients with cardiovascular disease treated with renin angiotensin aldosterone system inhibitors: coordinated by the Working Group on Cardiovascular Pharmacotherapy of the European Society of Cardiology. Eur. Heart J. Cardiovasc. Pharmacother. 4, 180–188. doi: 10.1093/ehjcvp/pvy015, PMID: 29726985 PMC13588647

[B136] Sahu D. Ganguly T. Mann A. Gupta Y. Nynatten L. R. V. Fraser D. D. (2025). Emerging technologies for exploring the cellular mechanisms in vascular diseases. Int. J. Mol. Sci. 27 (1), 164. doi: 10.3390/ijms27010164, PMID: 41516041 PMC12785399

[B137] Sanghvi S. Szteyn K. Ponnalagu D. Sridharan D. Lam A. Hansra I. . (2022). Inhibition of BK(Ca) channels protects neonatal hearts against myocardial ischemia and reperfusion injury. Cell Death Discov. 8, 175. doi: 10.1038/s41420-022-00980-z, PMID: 35393410 PMC8989942

[B138] Sarwar C. M. Papadimitriou L. Pitt B. Piña I. Zannad F. Anker S. D. . (2016). Hyperkalemia in heart failure. J. Am. Coll. Cardiol. 68, 1575–1589. doi: 10.1016/j.jacc.2016.06.060, PMID: 27687200

[B139] Schotten U. Verheule S. Kirchhof P. Goette A. (2011). Pathophysiological mechanisms of atrial fibrillation: a translational appraisal. Physiol. Rev. 91, 265–325. doi: 10.1152/physrev.00031.2009, PMID: 21248168

[B140] Schupp T. Bertsch T. von Zworowsky M. Kim S. H. Weidner K. Rusnak J. . (2020). Prognostic impact of potassium levels in patients with ventricular tachyarrhythmias. Clin. Res. Cardiol. 109, 1292–1306. doi: 10.1007/s00392-020-01624-x, PMID: 32236716

[B141] Schwartz P. J. Crotti L. Insolia R. (2012). Long-QT syndrome: from genetics to management. Circ. Arrhythm Electrophysiol. 5, 868–877. doi: 10.1161/CIRCEP.111.962019, PMID: 22895603 PMC3461497

[B142] Sfairopoulos D. Arseniou A. Korantzopoulos P. (2021). Serum potassium and heart failure: association, causation, and clinical implications. Heart Fail Rev. 26, 479–486. doi: 10.1007/s10741-020-10039-9, PMID: 33098029

[B143] Shima N. Yamamura A. Fujiwara M. Amano T. Matsumoto K. Sekine T. . (2024). Up-regulated expression of two-pore domain K(+) channels, KCNK1 and KCNK2, is involved in the proliferation and migration of pulmonary arterial smooth muscle cells in pulmonary arterial hypertension. Front. Cardiovasc. Med. 11, 1343804. doi: 10.3389/fcvm.2024.1343804, PMID: 38410243 PMC10894933

[B144] Shimada H. Mizuno K. Kawakami K. (2026). Effect of sodium zirconium cyclosilicate approval on the management of acute hyperkalemia in Japan: Interrupted time series analysis. Am. J. Nephrol. 10, 1–12. doi: 10.1159/000550435, PMID: 41518630 PMC12900531

[B145] Sørensen M. V. Sausbier M. Ruth P. Seidler U. Riederer B. Praetorius H. A. . (2010). Adrenaline-induced colonic K+ secretion is mediated by KCa1.1 (BK) channels. J. Physiol. 588, 1763–1777. doi: 10.1113/jphysiol.2009.181933, PMID: 20351045 PMC2887993

[B146] Souza A. C. R. Vasconcelos A. R. Dias D. D. Komoni G. Name J. J. (2023). The integral role of magnesium in muscle integrity and aging: A comprehensive review. Nutrients 15 (24), 5127. doi: 10.3390/nu15245127, PMID: 38140385 PMC10745813

[B147] Statland J. M. Fontaine B. Hanna M. G. Johnson N. E. Kissel J. T. Sansone V. A. . (2018). Review of the diagnosis and treatment of periodic paralysis. Muscle Nerve. 57, 522–530. doi: 10.1002/mus.26009, PMID: 29125635 PMC5867231

[B148] Stavniichuk A. Pyrshev K. Zaika O. Tomilin V. N. Kordysh M. Lakk M. . (2023). TRPV4 expression in the renal tubule is necessary for maintaining whole body K(+) homeostasis. Am. J. Physiol. Renal Physiol. 324, F603–Ff16. doi: 10.1152/ajprenal.00278.2022, PMID: 37141145 PMC10281785

[B149] Stone M. S. Martyn L. Weaver C. M. (2016). Potassium intake, bioavailability, hypertension, and glucose control. Nutrients 8 (7), 444. doi: 10.3390/nu8070444, PMID: 27455317 PMC4963920

[B150] Sukrittanon S. Watanapa W. B. Ruamyod K. (2014). Ginsenoside Re enhances small-conductance Ca(2+)-activated K(+) current in human coronary artery endothelial cells. Life Sci. 115, 15–21. doi: 10.1016/j.lfs.2014.09.007, PMID: 25242515

[B151] Sun Y. Byon C. H. Yang Y. Bradley W. E. Dell’Italia L. J. Sanders P. W. . (2017). Dietary potassium regulates vascular calcification and arterial stiffness. JCI Insight 2 (19), e94920. doi: 10.1172/jci.insight.94920, PMID: 28978809 PMC5841863

[B152] Sun Z. Jiao J. Lu G. Liu R. Li Z. Sun Y. . (2023). Overview of research progress on the association of dietary potassium intake with serum potassium and survival in hemodialysis patients, does dietary potassium restriction really benefit hemodialysis patients? Front. Endocrinol. (Lausanne) 14, 1285929. doi: 10.3389/fendo.2023.1285929, PMID: 38093955 PMC10716210

[B153] Suzumoto Y. Zucaro L. Iervolino A. Capasso G. (2023). Kidney and blood pressure regulation-latest evidence for molecular mechanisms. Clin. Kidney J. 16, 952–964. doi: 10.1093/ckj/sfad015, PMID: 37261007 PMC10229285

[B154] Tamargo J. Caballero R. Gómez R. Valenzuela C. Delpón E. (2004). Pharmacology of cardiac potassium channels. Cardiovasc. Res. 62, 9–33. doi: 10.1016/j.cardiores.2003.12.026, PMID: 15023549

[B155] Tan X. F. Qin T. Li N. Yang Y. G. Zheng J. H. Xie L. . (2019). High-potassium preconditioning enhances tolerance to focal cerebral ischemia-reperfusion injury through anti-apoptotic effects in male rats. J. Neurosci. Res. 97, 1253–1265. doi: 10.1002/jnr.24483, PMID: 31240758

[B156] Tetti M. Monticone S. Burrello J. Matarazzo P. Veglio F. Pasini B. . (2018). Liddle syndrome: review of the literature and description of a new case. Int. J. Mol. Sci. 19 (3), 812. doi: 10.3390/ijms19030812, PMID: 29534496 PMC5877673

[B157] Todkar A. Picard N. Loffing-Cueni D. Sorensen M. V. Mihailova M. Nesterov V. . (2015). Mechanisms of renal control of potassium homeostasis in complete aldosterone deficiency. J. Am. Soc. Nephrol.: JASN 26, 425–438. doi: 10.1681/ASN.2013111156, PMID: 25071088 PMC4310654

[B158] Tsao C. W. Aday A. W. Almarzooq Z. I. Anderson C. A. M. Arora P. Avery C. L. . (2023). Heart disease and stroke statistics-2023 update: A report from the american heart association. Circulation 147, e93–e621. doi: 10.1161/CIR.0000000000001123, PMID: 36695182 PMC12135016

[B159] Tsuji H. Venditti F. J. Jr. Evans J. C. Larson M. G. Levy D. (1994). The associations of levels of serum potassium and magnesium with ventricular premature complexes (the Framingham Heart Study). Am. J. Cardiol. 74, 232–235. doi: 10.1016/0002-9149(94)90362-X, PMID: 7518645

[B160] Udelson J. E. Selker H. P. Braunwald E. (2022). Glucose-insulin-potassium therapy for acute myocardial infarction: 50 years on and time for a relook. Circulation 146, 503–505. doi: 10.1161/CIRCULATIONAHA.121.058740, PMID: 35969651

[B161] Veiras L. C. Han J. Ralph D. L. McDonough A. A. (2016). Potassium supplementation prevents sodium chloride cotransporter stimulation during angiotensin II hypertension. Hypertension 68, 904–912. doi: 10.1161/HYPERTENSIONAHA.116.07389, PMID: 27600183 PMC5016236

[B162] Vera-Zambrano A. Lago-Docampo M. Gallego N. Franco-Gonzalez J. F. Morales-Cano D. Cruz-Utrilla A. . (2023). Novel loss-of-function KCNA5 variants in pulmonary arterial hypertension. Am. J. Respir. Cell Mol. Biol. 69, 147–158. doi: 10.1165/rcmb.2022-0245OC, PMID: 36917789

[B163] Verkerk A. O. Wilders R. (2014). Pacemaker activity of the human sinoatrial node: effects of HCN4 mutations on the hyperpolarization-activated current. Europace 16, 384–395. doi: 10.1093/europace/eut348, PMID: 24569893

[B164] Wan J. Chen M. Wang Z. Everett T. H. Rubart-von der Lohe M. Shen C. . (2019). Small-conductance calcium-activated potassium current modulates the ventricular escape rhythm in normal rabbit hearts. Heart Rhythm. 16, 615–623. doi: 10.1016/j.hrthm.2018.10.033, PMID: 30445170 PMC6443460

[B165] Wang J. Bai J. Duan P. Wang H. Li Y. Zhu Q. (2021). Kir6.1 improves cardiac dysfunction in diabetic cardiomyopathy via the AKT-FoxO1 signalling pathway. J. Cell Mol. Med. 25, 3935–3949. doi: 10.1111/jcmm.16346, PMID: 33547878 PMC8051713

[B166] Wang Z. Subramanya A. R. Satlin L. M. Pastor-Soler N. M. Carattino M. D. Kleyman T. R. (2013). Regulation of large-conductance Ca2+-activated K+ channels by WNK4 kinase. Am. J. Physiol. Cell Physiol. 305, C846–C853. doi: 10.1152/ajpcell.00133.2013, PMID: 23885063 PMC3798677

[B167] Wang X. Tian X. Shen H. Zhang X. Xie L. Chen M. (2023). Moderate hyperkalemia regulates autophagy to reduce cerebral ischemia-reperfusion injury in a CA/CPR rat model. Brain Sci. 13 (9), 1285. doi: 10.3390/brainsci13091285, PMID: 37759886 PMC10526941

[B168] Weiner I. D. Wingo C. S. (1997). Hypokalemia--consequences, causes, and correction. J. Am. Soc. Nephrol. 8, 1179–1188. doi: 10.1681/ASN.V871179, PMID: 9219169

[B169] Weiss J. N. Qu Z. Shivkumar K. (2017). Electrophysiology of hypokalemia and hyperkalemia. Circ. Arrhythm Electrophysiol. 10 (3), e004667. doi: 10.1161/CIRCEP.116.004667, PMID: 28314851 PMC5399982

[B170] Welling P. A. Ho K. (2009). A comprehensive guide to the ROMK potassium channel: form and function in health and disease. Am. J. Physiol. Renal Physiol. 297, F849–F863. doi: 10.1152/ajprenal.00181.2009, PMID: 19458126 PMC2775575

[B171] Wen D. Cornelius R. J. Rivero-Hernandez D. Yuan Y. Li H. Weinstein A. M. . (2014a). Relation between BK-α/β4-mediated potassium secretion and ENaC-mediated sodium reabsorption. Kidney Int. 86, 139–145. doi: 10.1038/ki.2014.14, PMID: 24573316 PMC4077913

[B172] Wen D. Cornelius R. J. Sansom S. C. (2014b). Interacting influence of diuretics and diet on BK channel-regulated K homeostasis. Curr. Opin. Pharmacol. 15, 28–32. doi: 10.1016/j.coph.2013.11.001, PMID: 24721651 PMC3984455

[B173] Wester P. O. (1992). Electrolyte balance in heart failure and the role for magnesium ions. Am. J. Cardiol. 70, 44c–49c. doi: 10.1016/0002-9149(92)91357-A, PMID: 1414894

[B174] Whelton P. K. (2014). Sodium, potassium, blood pressure, and cardiovascular disease in humans. Curr. Hypertens. Rep. 16, 465. doi: 10.1007/s11906-014-0465-5, PMID: 24924995

[B175] Whittaker D. G. Colman M. A. Ni H. Hancox J. C. Zhang H. (2018). Human atrial arrhythmogenesis and sinus bradycardia in KCNQ1-linked short QT syndrome: insights from computational modelling. Front. Physiol. 9, 1402. doi: 10.3389/fphys.2018.01402, PMID: 30337886 PMC6180159

[B176] Wiedmann F. Frey N. Schmidt C. (2021). Two-pore-domain potassium (K(2P)-) channels: cardiac expression patterns and disease-specific remodelling processes. Cells 10 (11), 2914. doi: 10.3390/cells10112914, PMID: 34831137 PMC8616229

[B177] Wu P. Gao Z. X. Zhang D. D. Duan X. P. Terker A. S. Lin D. H. . (2020). Effect of angiotensin II on ENaC in the distal convoluted tubule and in the cortical collecting duct of mineralocorticoid receptor deficient mice. J. Am. Heart Assoc. 9, e014996. doi: 10.1161/JAHA.119.014996, PMID: 32208832 PMC7428622

[B178] Wu H. Huang R. Fan J. Luo N. Yang X. (2022). Low potassium disrupt intestinal barrier and result in bacterial translocation. J. Transl. Med. 20, 309. doi: 10.1186/s12967-022-03499-0, PMID: 35794599 PMC9258207

[B179] Xie Y. Mossavar-Rahmani Y. Chen Y. Abramowitz M. K. Chen W. (2023). Association of dietary potassium intake with abdominal aortic calcification and pulse pressure in US adults. J. Ren. Nutr. 33, 657–665. doi: 10.1053/j.jrn.2023.06.003, PMID: 37302720 PMC10528025

[B180] Xiong Q. Cao Q. Zhou Q. Xie J. Shen Y. Wan R. . (2015). Arrhythmogenic cardiomyopathy in a patient with a rare loss-of-function KCNQ1 mutation. J. Am. Heart Assoc. 4, e001526. doi: 10.1161/JAHA.114.001526, PMID: 25616976 PMC4330077

[B181] Xu C. Chen Y. Wang F. Xie S. Yang T. (2021). Soluble (Pro)Renin receptor as a negative regulator of NCC (Na(+)-cl(-) cotransporter) activity. Hypertension 78, 1027–1038. doi: 10.1161/HYPERTENSIONAHA.121.16981, PMID: 34495675 PMC9212213

[B182] Xu S. Ilyas I. Little P. J. Li H. Kamato D. Zheng X. . (2021). Endothelial dysfunction in atherosclerotic cardiovascular diseases and beyond: from mechanism to pharmacotherapies. Pharmacol. Rev. 73, 924–967. doi: 10.1124/pharmrev.120.000096, PMID: 34088867

[B183] Xu C. Lu A. Wang H. Fang H. Zhou L. Sun P. . (2017). (Pro)Renin receptor regulates potassium homeostasis through a local mechanism. Am. J. Physiol. Renal Physiol. 313, F641–Ff56. doi: 10.1152/ajprenal.00043.2016, PMID: 27440776 PMC5625102

[B184] Yang L. Frindt G. Palmer L. G. (2010). Magnesium modulates ROMK channel-mediated potassium secretion. J. Am. Soc. Nephrol. 21, 2109–2116. doi: 10.1681/ASN.2010060617, PMID: 21030597 PMC3014024

[B185] Yin D. Hsieh Y. C. Tsai W. C. Wu A. Z. Jiang Z. Chan Y. H. . (2017). Role of apamin-sensitive calcium-activated small-conductance potassium currents on the mechanisms of ventricular fibrillation in pacing-induced failing rabbit hearts. Circ. Arrhythm Electrophysiol. 10, e004434. doi: 10.1161/CIRCEP.116.004434, PMID: 28213506 PMC5351779

[B186] Zareba W. Cygankiewicz I. (2008). Long QT syndrome and short QT syndrome. Prog. Cardiovasc. Dis. 51, 264–278. doi: 10.1016/j.pcad.2008.10.006, PMID: 19026859

[B187] Zhang W. Lei X. J. Wang Y. F. Wang D. Q. Yuan Z. Y. (2016). Role of Kir2.1 in human monocyte-derived foam cell maturation. J. Cell Mol. Med. 20, 403–412. doi: 10.1111/jcmm.12705, PMID: 26689595 PMC4759473

[B188] Zhang Q. Liu L. Hu Y. Shen L. Li L. Wang Y. (2022). Kv1.3 Channel Is Involved In Ox-LDL-induced Macrophage Inflammation Via ERK/NF-κB signaling pathway. Arch. Biochem. Biophys. 730, 109394. doi: 10.1016/j.abb.2022.109394, PMID: 36100082

[B189] Zhang M. Liu Q. Meng H. Duan H. Liu X. Wu J. . (2024). Ischemia-reperfusion injury: molecular mechanisms and therapeutic targets. Signal Transduct. Target Ther. 9, 12. doi: 10.1038/s41392-023-01688-x, PMID: 38185705 PMC10772178

[B190] Zhu Y. R. Jiang X. X. Zhang D. M. (2019). Critical regulation of atherosclerosis by the KCa3.1 channel and the retargeting of this therapeutic target in in-stent neoatherosclerosis. J. Mol. Med. (Berl) 97, 1219–1229. doi: 10.1007/s00109-019-01814-9, PMID: 31254004

[B191] Zicha S. Xiao L. Stafford S. Cha T. J. Han W. Varro A. . (2004). Transmural expression of transient outward potassium current subunits in normal and failing canine and human hearts. J. Physiol. 561, 735–748. doi: 10.1113/jphysiol.2004.075861, PMID: 15498806 PMC1665387

[B192] Zillich A. J. Garg J. Basu S. Bakris G. L. Carter B. L. (2006). Thiazide diuretics, potassium, and the development of diabetes: a quantitative review. Hypertension. 48, 219–224. doi: 10.1161/01.HYP.0000231552.10054.aa, PMID: 16801488

